# Alleviation of preeclampsia-like symptoms through PlGF and eNOS regulation by hypoxia- and NF-κB-responsive miR-214-3p deletion

**DOI:** 10.1038/s12276-024-01237-8

**Published:** 2024-06-03

**Authors:** Suji Kim, Sungbo Shim, Jisoo Kwon, Sungwoo Ryoo, Junyoung Byeon, Jungwoo Hong, Jeong-Hyung Lee, Young-Guen Kwon, Ji-Yoon Kim, Young-Myeong Kim

**Affiliations:** 1 Department of Molecular and Cellular Biochemistry, School of Medicine, Kangwon National University, Chuncheon, 24341 Republic of Korea; 2 Department of Biochemistry, Chungbuk National University, Cheongju, 28644 Republic of Korea; 3 Department of Anesthesiology and Pain Medicine, Hanyang University Hospital, Hanyang University College of Medicine, Seoul, 04763 Republic of Korea; 4 Department of Biological Sciences, Kangwon National University, Chuncheon, 24341 Republic of Korea; 5 Department of Biochemistry, College of Natural Sciences, Kangwon National University, Chuncheon, 24341 Republic of Korea; 6 Advanced Institute of Technology, Curacle Co. Ltd, Seoul, 06694 Republic of Korea

**Keywords:** Mechanisms of disease, Embryonic induction, Pre-eclampsia, Predictive markers

## Abstract

Preeclampsia is caused by placental hypoxia and systemic inflammation and is associated with reduced placental growth factor (PlGF) and endothelial nitric oxide synthase (eNOS) levels. The molecular signaling axes involved in this process may play a role in the pathogenesis of preeclampsia. Here, we found that hypoxic exposure increased hypoxia-inducible factor-1α (HIF-1α)/Twist1-mediated miR-214-3p biogenesis in trophoblasts, suppressing PlGF production and trophoblast invasion. TNF-α stimulation increased NF-κB-dependent miR-214-3p expression in endothelial cells, impairing eNOS expression and causing endothelial dysfunction. Synthetic miR-214-3p administration to pregnant mice decreased PlGF and eNOS expression, resulting in preeclampsia-like symptoms, including hypertension, proteinuria, and fetal growth restriction. Conversely, miR-214-3p deletion maintained the PlGF and eNOS levels in hypoxic pregnant mice, alleviating preeclampsia-like symptoms and signs. These findings provide new insights into the role of HIF-1/Twist1- and NF-κB-responsive miR-214-3p-dependent PlGF and eNOS downregulation in the pathogenesis of preeclampsia and establish miR-214-3p as a therapeutic or preventive target for preeclampsia and its complications.

## Hypoxia-induced miR-214-3p: key player in preeclampsia pathogenesis

This research investigates how a molecule, miR-214-3p, impacts the occurrence of preeclampsia - a pregnancy issue marked by high blood pressure and organ damage. Scientists discovered that miR-214-3p produced more when oxygen levels are low, suppresses two other molecules, PlGF and eNOS, essential for a healthy pregnancy. In mouse experiments, artificially increasing miR-214-3p led to preeclampsia-like symptoms, while mice without miR-214-3p alleviated these symptoms in low oxygen. This suggests miR-214-3p could be a focus for preeclampsia prevention or treatment. Further studies are required to confirm this. “This summary was initially drafted using artificial intelligence, then revised and fact-checked by the author.”

## Introduction

The placenta is a unique organ that separates the maternal and fetal compartments and mediates the exchange of nutrients, oxygen, and waste products between the maternal and fetal circulation, thereby ensuring proper fetal growth and development^1^. For increased efficacy of these processes, maternal uterine spiral arteries undergo extensive remodeling in the early stages of pregnancy by invading fetal trophoblasts and replacing endothelial and smooth muscle cells in the arterial wall^2^. Thus, inadequate or inappropriate spiral artery remodeling leads to abnormal placental blood flow and subsequent pregnancy complications, including intrauterine growth retardation and preeclampsia (PE)^1^.

PE is a pregnancy-specific disease characterized by new-onset hypertension (≥140/90 mmHg) and proteinuria (≥300 mg/24 h) after 20 weeks of gestation. Although the etiology of PE is not well understood, its pathogenesis originates from placental hypoxia, which causes maternal systemic inflammation, eventually resulting in vascular and renal vascular dysfunction^1,3^. Placental hypoxia activates hypoxia-inducible factor-1α (HIF-1α) signaling, which upregulates the expression of antiangiogenic factors, such as soluble fms-like tyrosine kinase-1 (sFlt-1) and soluble endoglin (sEng), and downregulates that of proangiogenic placental growth factor (PlGF)^4–6^, thereby increasing the sFlt-1/PlGF ratio and impairing endothelial cell function and trophoblast invasion^7^. Nevertheless, HIF-1α promotes PlGF expression in nonplacental tissues, particularly in nontrophoblast cells^8,9^. Therefore, hypoxia differentially regulates PlGF expression in trophoblasts and nontrophoblasts. Elucidating this mechanism may provide important clues regarding the pathogenesis of PE.

Chronic placental hypoxia also stimulates the expression and secretion of NF-κB-responsive inflammatory cytokines, including TNF-α and IL-1β, into the maternal circulatory system; these inflammatory cytokines are considered important risk factors for endothelial cell dysfunction in women with PE^3,10,11^. Consistent with the changes in cytokine levels, the expression of several inflammation-responsive miRNAs is increased in the serum of patients with PE and negatively regulates the translation of target genes involved in the pathogenesis of PE^12,13^. Indeed, several studies have demonstrated that NF-κB-induced miR-31-5p and miR-155-5p levels are elevated in the circulatory system of women with PE^13–16^, and these molecules impair angiogenesis and vasorelaxation through downregulation of endothelial nitric oxide synthase (eNOS) and soluble guanylate cyclase expression by targeting the 3′-untranslated regions (3′-UTRs) of their mRNAs in human vascular endothelial and smooth muscle cells^14–16^. In addition, miR-214-3p expression is upregulated in response to HIF-1α and NF-κB^17,18^ and inhibits eNOS and PlGF expression^19,20^. Thus, circulating miR-214-3p levels are elevated and inversely correlated with serum nitric oxide (NO) and PlGF levels in patients with PE^13^. These findings suggest that miRNAs play important roles in placental and vascular dysfunction. However, their function in the pathogenesis of PE has not yet been elucidated.

Based on the available evidence, we hypothesized that upregulation of miRNA expression under hypoxic and/or inflammatory conditions leads to functional impairment of trophoblasts and endothelial cells by downregulating PlGF and eNOS expression, respectively, resulting in PE-like features. Therefore, in this study, we investigated the ability of miR-214-3p deletion or inhibition to downregulate placental PlGF and vascular eNOS expression and induce PE-like symptoms in a pregnant mouse model. Our results provide new insights into the role of miR-214-3p in the pathogenesis of PE.

## Materials and methods

### Materials

The antibodies, miRNA primers, ELISA kits, recombinant proteins, and cell culture media and supplements used in this study are described in the Supplementary Information.

### Cell culture and treatment

HTR-8/SVneo cells (ATCC® CRL3271™, Gaithersburg, MD, USA), BeWo cells (10098, Korea Cell Line Bank, Seoul, South Korea), HUVECs (8000, ScienCell Research Labs, San Diego, CA, USA), and human aortic smooth muscle cells (HASMCs; 6110, ScienCell Research Labs) were cultured in RPMI 1640 medium, endothelial cell medium, and smooth muscle cell medium as described previously^14,16^. MLECs were isolated from 7- to 8-week-old female C57BL/6 mice using an immunobead protocol as described previously^14^. Cells were transfected with siRNA (80 nM), miRNA (80 nM), or plasmid DNA (2 μg/ml) using Lipofectamine RNAiMAX or Lipofectamine 3000. After recovery in fresh medium for 24 h, some cells were pretreated with Bay 11-7082 (5 μM) or IDF-11774 (10 μM) for 30 min, followed by culture in a normoxic incubator or hypoxic incubator chamber (2% O_2_, 5% CO_2_, and 93% N_2_) or treatment with TNF-α (10 ng/ml) for either 24 h or the durations indicated in the figures.

### Generation of miR-214-3p KO mice

MiR-214-3p KO mice were generated using the CRISPR/Cas9 system. The detailed procedures are described in the Supplementary Information.

### Animal experiments

Seven- to eight-week-old female WT and miR-214-3p KO mice (C57BL/6) were mated with male mice of the corresponding genotypes. For in vivo functional investigation of synthetic miR-214-3p, pregnant WT mice were randomly divided into two groups, intraperitoneally anesthetized with ketamine (90 mg/kg) and xylazine (10 mg/kg), and implanted with an Alzet osmotic pump (1007D, Alza Corp., Palo Alto, CA, USA) in the peritoneal cavity according to the manufacturer’s protocol. The mice were continuously infused with locked nucleic acid control miRNA (YM00479903, QIAGEN, Hilden, Germany), miR-214-3p mimic (YM00472980, QIAGEN), or miR-214-3p mimic plus recombinant mouse PlGF-2 (rmPlGF-2; 465-PL, carrier-free, R&D Systems) at a rate of 0.5 μl/h from GD 9.5 to GD 17, and each mouse received miRNA (10 μg/day) or rmPlGF-2 (1.2 μg/day). For analysis the pathogenesis of PE, pregnant WT and miR-214-3p KO mice were randomly divided into hypoxic (9.5% O_2_) and normoxic or control (21% O_2_) groups. The mice assigned to the hypoxic group were placed in a hermetically sealed chamber (Coy Laboratory Products, Inc., Grass Lake, MI, USA) with continuous control of O_2_ concentrations (calibrated for 9.5% O_2_, 5% CO_2_, and 85.5% N_2_), temperature (24–26 °C), and humidity (30–70%) from GD 9.5 to GD 17.5. Following blood collection by heart puncture under isoflurane anesthesia, the mice were sacrificed by cervical dislocation, and the fetuses, placentas, kidneys, and urine were collected. All specimens were immediately analyzed or stored at −80 °C until use.

### Measurement of blood pressure and vascular relaxation

After the mice were maintained under normoxic or hypoxic conditions, blood pressure was measured 3 times by setting 10 cycles per set after 5 acclimation cycles using the noninvasive CODA-HT4 tail-cuff system (Kent Scientific, Torrington, CT, USA) according to the manufacturer’s protocol. The vasorelaxation responses of isolated aortic vessels to Ach were assayed using a multiwire myography system (DMT-620M, Aarhus, Denmark) as previously described^16^. Some vessels were treated in vitro with mouse TNF-α (10 ng/ml), Bay 11-7082 (5 μM), or a miR-214-3p inhibitor (80 nM) for 18 h before the vascular relaxation assay.

### MiRNA profiling and quantitative real-time PCR (qRT‒PCR) analysis

The expression of miRNAs in HTR-8/SVneo cells exposed to normoxia or hypoxia for 24 h was analyzed at Macrogen (Seoul, Korea) using the Affymetrix miRNA expression microarray version 3.0 (902017). Relative miRNA and mRNA levels were determined using qRT‒PCR as described previously^13,18^, and detailed procedures are provided in the Supplementary Information.

### CM preparation and treatment

HTR-8/SVneo cells were transfected with control miRNA or an miR-214-3p inhibitor for 8 h using Lipofectamine RNAiMAX and incubated with fresh medium in a normoxic incubator or hypoxic incubator for another 24 h. Culture media were collected and centrifuged at 4 °C and 12,000 × *g* for 10 min in a microcentrifuge, and the supernatants were used as CM. CM was premixed with an anti-VEGF neutralizing antibody (5 μg/ml) or an anti-PlGF neutralizing antibody (5 μg/ml) for 30 min at 37 °C and used to assay endothelial cell angiogenesis and trophoblast migration and invasion. Some cells were transfected with 80 nM control or *Vegfr1* siRNA using Lipofectamine RNAiMAX before culture in CM.

### In vitro endothelial cell angiogenesis and trophoblast migration/invasion assays

For the in vitro angiogenesis assay, endothelial cell migration and tube formation were assessed as previously described^14^. Trophoblast migration and invasion were assessed by scratch-wound assay and Boyden chamber assay, respectively. The detailed procedures are described in the Supplementary Information.

### Histology and immunohistochemistry

Paraffin-embedded kidney and placental tissues were sectioned at a thickness of 5 μm and stained with H&E and target-specific antibodies, followed by analysis under an Olympus IX71 microscope. The detailed procedures are described in the Supplementary Information.

### Promoter prediction and luciferase reporter activity

Bioinformatics analysis and prediction of putative transcription factor-binding sites in the promoter sequences were performed using PROMO software (version 8.3 of TRANSFAC). HTR-8/SVneo cells, HUVECs, or HASMCs were transfected with siRNAs (80 nM) or miRNAs (80 nM) as well as 2 μg/ml of mock vector, psiCHECK-2-WT or mutant *PlGF* 3′-UTR (human, 876 bp), psiCHECK-2-*eNOS* 3′-UTR (mouse, 470 bp; human, 430 bp), pGL3-*PlGF* promoter (human, ~1.9 kb), pGL3-WT or truncated *Dnm3os* (miR-199a/214) promoter (human, 2,286 kb), pcDNA3.1-mutant *HIF1A* (P402A/P564G, double mutation to prevent proline hydroxylation and its degradation under normoxia^21^), or pGL4.74 vector (*Renilla* luciferase reporter plasmid) using Lipofectamine 3000 or Lipofectamine RNAiMAX. Cells were treated with TNF-α (10 ng/ml) or exposed to normoxia or hypoxia in the presence or absence of Bay 11-7082 (5 μM) or IDF-11774 (10 μM). Firefly and *Renilla* luciferase activities were assayed using a dual-luciferase reporter assay kit (E1960, Promega), and firefly luciferase activity was normalized to the *Renilla* luciferase signal.

### Other biochemical analyses

Western blot analysis was performed using cell or tissue lysates (30 μg of protein) as previously described^11^. The levels of PlGF (DPG00 for human; MP200 for mouse), VEGF (MMV00), sFlt-1 (MVR100), sEng (MNDG00), TNF-α (MTA00B), IL-1β (MLB00C), IL-6 (M6000B), and cGMP (KGE003) were analyzed in cell culture media, sera, or tissue lysates using Quantikine ELISA kits (R&D Systems) according to the manufacturer’s protocol. Urinary albumin and creatinine levels were determined using an albumin ELISA kit (80630) and a creatinine assay kit (80350), respectively, purchased from Crystal Chem (Elk Grove, IL, USA). Intracellular NO levels were measured in cultured endothelial cells and aortic vessels under a fluorescence microscope using the NO probe DAF-FM diacetate^14,22^.

### Statistics and reproducibility

No statistical methods were used to determine the sample size. The experiments were randomized, the investigators were blinded to allocation during the experiments and outcome analysis, and no samples or animals were excluded from the analyses. All the data are presented as the mean ± standard error of the mean (SEM) of at least four independent experiments. Statistical significance was determined using an unpaired two-tailed *t* test, a multiple *t* test, or one-way or two-way analysis of variance (ANOVA), followed by the Holm–Sidak’s multiple comparison test, depending on the experimental variables or analyzed groups. Statistical analyses were performed using Prism (version 6.0; GraphPad Software, Inc.). Statistical significance was set at *P* ≤ 0.05.

## Results

### Hypoxia downregulates PlGF expression in trophoblasts via miR-214-3p biogenesis

**Fig. 1:**
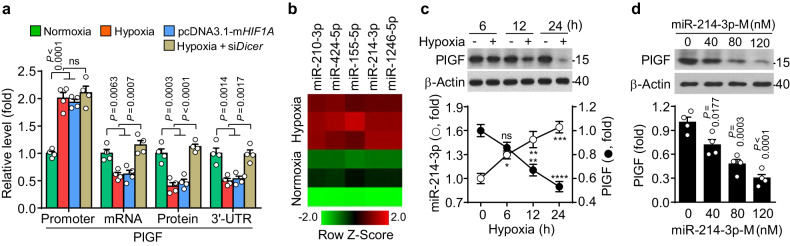
Hypoxia increases miR-214-3p biogenesis, which, in turn, inhibits PlGF expression in trophoblasts. **a** HTR-8/SVneo cells were transfected with empty (mock) vector, pcDNA3.1-mutant *HIF1A* (m*HIF1A*), pGL3-*PlGF* promoter, psiCHECK-2-*PlGF* 3′-UTR, or *Dicer* siRNA (si*Dicer*) and subjected to normoxia or hypoxia for 24 h. *PlGF* mRNA and protein levels were determined using qRT‒PCR and western blotting, respectively (*n* = 4). *PlGF* promoter and mRNA 3′-UTR activities were determined in cell lysates using a luciferase activity assay (*n* = 4). **b** The expression profile of miRNAs was assessed in HTR-8/SVneo cells exposed to normoxia or hypoxia for 24 h using a microarray (*n* = 3). **c** The expression of miR-214-3p and PlGF in HTR-8/SVneo cells exposed to normoxia or hypoxia was determined using qRT‒PCR and western blotting, respectively (*n* = 4). **d** HTR-8/SVneo cells were transfected with miR-214-3p mimics (miR-214-3p-M) for 24 h. PlGF levels were determined using western blotting (*n* = 4). The data are presented as the mean ± SEM. Statistical significance was determined using one-way ANOVA, followed by the Holm–Sidak’s multiple comparisons test. ns not significant.

As PlGF expression is downregulated in the placenta under hypoxic conditions associated with the pathogenesis of PE^6,7^, we first investigated the mechanism by which hypoxia suppresses PlGF expression in trophoblasts. Compared with normoxic conditions, hypoxic treatment or overexpression of mutant HIF-1α (P402A/P564G) significantly increased the transcriptional activity of the *PlGF* promoter, which contains two hypoxia response elements (HREs)^19^, but reduced *PlGF* mRNA and protein levels and the functional activity of its 3′-UTR in the human trophoblast cell line HTR-8/SVneo (Fig. 1a and Supplementary Fig. 1a–c). This result suggests that hypoxia stimulates the transcription of *PlGF* but decreases its translation, probably through the biogenesis of HIF-1-responsive miRNAs that target the 3′-UTR of *PlGF* mRNA. To verify this hypothesis, we examined the effects of siRNA-mediated knockdown of *Dicer*, an endoribonuclease gene that governs the maturation of precursor miRNAs, on the hypoxia-mediated regulation of PlGF expression^23^. *Dicer* knockdown did not affect *PlGF* promoter activity and prevented the hypoxia-mediated reduction in *PlGF* 3′-UTR activity; however, its knockdown returned *PlGF* mRNA and protein expression to the normoxic control level but not to the hypoxia-induced level of *PlGF* promoter activity in HTR-8/SVneo cells (Fig. 1a and Supplementary Fig. 1d), suggesting that hypoxia suppresses PlGF expression in miRNA-dependent and miRNA-independent manners. Notably, we identified several miRNAs (miR-155-5p, miR-210-3p, miR-214-3p, miR-424-5p, and miR-1246-5p) that were potentially upregulated in response to hypoxia using mRNA microarray analysis (Fig. 1b). With the TargetScan algorithm, miR-214-3p was predicted to target the 3′-UTR of human *PlGF* mRNA at three different regions (71–77, 111–118, and 415–421 nt) (Supplementary Fig. 2a). The two proximal regions are highly conserved in humans, nonhuman primates, and rodents, whereas the distal region is conserved only in humans and nonhuman primates (Supplementary Fig. 2b). Hypoxic treatment increased miR-214-3p levels in a time-dependent manner, which was inversely correlated with PlGF levels (Fig. 1c). Transfection with miR-214-3p mimics decreased PlGF levels in a dose-dependent manner (Fig. 1d), and treatment with an miR-214-3p inhibitor reversed the hypoxia-induced suppression of PlGF expression (Supplementary Fig. 1e). These data suggest that hypoxia downregulates PlGF expression in trophoblasts by increasing miR-214-3p biogenesis.

### Hypoxia increases miR-214-3p biogenesis through the HIF-1/Twist1 axis

**Fig. 2:**
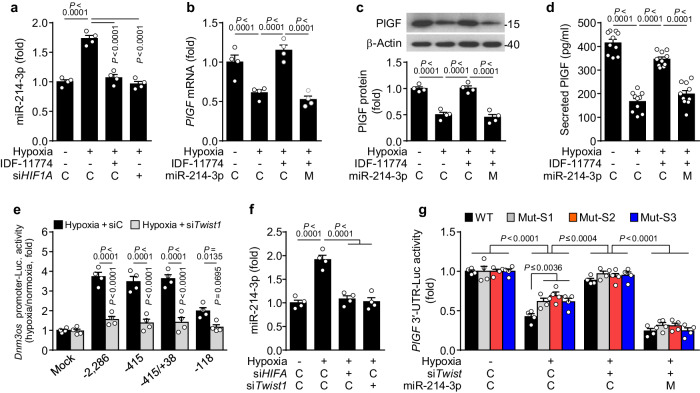
HIF-1α-responsive miR-214-3p inhibits PlGF expression by targeting its mRNA 3′-UTR. **a**–**d** HTR-8/SVneo cells were transfected with control (C), *HIF1A* siRNA (si*HIF1A*), control miRNA (C), or miR-214-3p mimics (M), followed by exposure to normoxia or hypoxia in the presence or absence of IDF-11774 for 24 h. The levels of miR-214-3p (*n* = 4, **a**), *PlGF* mRNA (*n* = 4, **b**), PlGF protein (*n* = 4, **c**), and secreted PlGF (*n* = 10, **d**) were determined using qRT‒PCR, western blotting, or ELISA. **e** Cells were transfected with control siRNA (siC), *Twist1* siRNA (si*Twist1*), or pGL3-WT or -truncated *Dnm3os* promoter (see Supplementary Fig. 3i), followed by exposure to normoxia or hypoxia. Luciferase activity in the cell lysates was assayed using an enzyme assay kit and normalized to the signal under normoxic conditions (*n* = 4). **f** Cells were transfected with siRNA for control (C), *HIF1A*, or *Twist1*, followed by exposure to normoxia or hypoxia. MiR-214-3p levels were determined using qRT‒PCR (*n* = 4). **g** Cells were transfected with psiCHECK-2-WT or mutant *PlGF* 3′-UTRs (Mut-S1, Mut-S2, and Mut-S3 represent mutants at sites 1, 2, and 3, respectively; Supplementary Fig. 3k) in combination with control siRNA (C), *Twist1* siRNA, control miRNA (C), or miR-214-3p mimics (M), followed by exposure to normoxia or hypoxia. Luciferase reporter activities were determined in cell lysates using an enzyme assay kit (*n* = 4). The data are presented as the mean ± SEM. Statistical significance was determined using one-way (**a**–**d**, **f**) or two-way ANOVA (**e**, **g**), followed by the Holm–Sidak′s multiple comparisons test.

To investigate the role of hypoxia in miR-214-3p biogenesis and PlGF expression, we analyzed the transcriptional activity of the promoter of the long noncoding RNA dynamin-3 opposite strand (*Dnm3os*), which encodes the miR-199a/214 cluster. Hypoxia increased the levels of pre- and mature miR-214-3p and its cluster miR-199a-3p in HTR-8/SVneo cells, whereas their expression was blocked by treatment with the HIF-1α inhibitor IDF-11774 or *HIF1A* siRNA (Fig. 2a and Supplementary Fig. 3a, b). As expected, IDF-11774 treatment rescued the hypoxia-mediated decrease in PlGF expression and secretion in HTR-8/SVneo cells; however, these effects were blocked by transfection with miR-214 mimics (Fig. 2b–d). Similar results were observed for miR-214-3p biogenesis, PlGF expression, and PlGF secretion in the human choriocarcinoma cell line BeWo under hypoxic conditions (Supplementary Fig. 3c–f). These results suggest that hypoxia inhibits PlGF expression via HIF-1-dependent miR-214-3p biogenesis. However, computational analysis revealed a lack of HREs in the promoter region of the human and mouse *Dnm3os* genes (Supplementary Fig. 3g, h), indicating that hypoxia promotes miR-214-3p biogenesis in an indirect HIF-1-mediated manner. Notably, the human and mouse *Dnm3os* promoters contain five and three putative palindromic E-box motifs with the consensus sequence CANNTG, respectively, which are known to bind the HIF-1-responsive transcription factor Twist1^24,25^ (Supplementary Fig. 3g, h). A luciferase reporter assay demonstrated that the proximal region of the human *Dnm3os* promoter (nt −415/+38), which contains five putative E-box motifs, was most sensitive to hypoxia, but this sensitivity was blocked by *Twist1* knockdown (Fig. 2e and Supplementary Fig. 3i). We further examined whether Twist1 regulates the transcriptional activity of the human *Dnm3os* promoter using point mutations of two E-box motifs located between nt −248 and nt 0 (Supplementary Fig. 3j). Each mutation in both motifs significantly reduced *Dnm3os* promoter activity, and the double mutation further suppressed *Dnm3os* promoter activity (Supplementary Fig. 3j). Consequently, hypoxia increased HIF-1α, Twist1, and miR-214-3p expression and suppressed PlGF expression, and all these changes were blocked by *HIF1A* knockdown. However, *Twist1* knockdown reversed hypoxia-mediated miR-214-3p biogenesis and PlGF downregulation but did not affect HIF-1α expression (Fig. 2f and Supplementary Fig. 3k). This finding indicates that hypoxia increases miR-214-3p expression via the HIF-1/Twist1 axis and subsequently downregulates PlGF expression. To verify whether *PlGF* mRNA is a bona fide target of miR-214-3p, we assayed the reporter activities of the human wild-type (WT) *PlGF* mRNA 3′-UTR and its mutants at each of three putative seed binding sites for miR-214-3p (Supplementary Fig. 3l). Hypoxia inhibited WT *PlGF* 3′-UTR-luciferase activity but did so less effectively with the three mutant *PlGF* 3′-UTR-reporter activities, with all the inhibitory effects reversed by *Twist1* knockdown; however, these recovery effects were strongly suppressed by transfection with miR-214-3p mimics (Fig. 2g). Collectively, these results suggest that hypoxia increases miR-214-3p biogenesis in a HIF-1/Twist1-dependent manner, thereby inhibiting PlGF expression in trophoblasts.

### Hypoxia increases PlGF expression but not miR-214 biogenesis in nontrophoblast cells

Next, we examined the effect of hypoxia on PlGF expression and miR-214-3p biogenesis in nontrophoblast cells. Hypoxic treatment increased *PlGF* promoter activity and *PlGF* mRNA and protein levels in human umbilical vein endothelial cells (HUVECs) and human aortic smooth muscle cells (HASMCs), and these effects were blocked by treatment with IDF-11774 but not by transfection with *Twist1* siRNA (Supplementary Fig. 4a–c). Notably, hypoxia did not increase miR-214-3p expression in vascular cells, and its levels were not altered by treatment with IDF-11774 or *Twist1* siRNA (Supplementary Fig. 4d). Consequently, hypoxia did not significantly decrease the reporter activity of the WT or mutant *PlGF* 3′-UTRs; moreover, these activities were not altered by *Twist* knockdown but were effectively inhibited by miR-214-3p mimics (Supplementary Fig. 4e). These results suggest that hypoxia increases PlGF expression without affecting miR-214-3p biogenesis in HUVECs and HASMCs in a HIF-1-dependent manner.

### The miR-214-3p/PlGF axis inhibits trophoblast invasion and angiogenesis

**Fig. 3:**
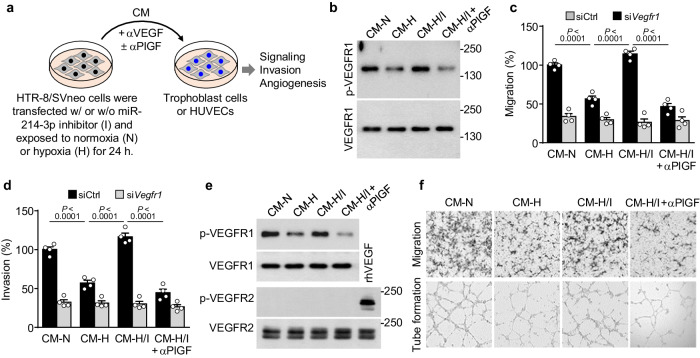
Hypoxic trophoblast-derived conditioned medium (CM) inhibits trophoblast invasion and endothelial cell angiogenesis. **a** Schematic diagram of CM preparation. HTR-8/SVneo cells were transfected with a miR-214-3p inhibitor and exposed to normoxia or hypoxia. CM was preincubated with an anti-VEGF neutralizing antibody (αVEGF). The biological effects of CM on trophoblasts and HUVECs were assessed in the presence or absence of an anti-PlGF neutralizing antibody (αPlGF). CM-N, CM from normoxic cells; CM-H, CM from hypoxic cells; CM-H/I, CM from hypoxic cells transfected with a miR-214-3p inhibitor. **b**–**d** HTR-8/SVneo cells were transfected with control (siCtrl) or *Vegfr1* siRNA (si*Vegfr1*) and incubated with CM. VEGFR1 phosphorylation (**b**), trophoblast migration (*n* = 4, **c**), and trophoblast invasion (*n* = 4, **d**) were determined using western blotting, the scratch-wound assay, and the Boyden chamber assay, respectively. **e**, **f** HUVECs were incubated with CM or treated with rhVEGF (10 ng/ml) as a positive control. **e** Phosphorylated VEGFR1 and VEGFR2 levels were determined using western blotting. **f** HUVEC migration and tube formation were determined using a Boyden chamber and growth factor-reduced Matrigel, respectively. The data are presented as the mean ± SEM. Statistical significance was determined using one-way ANOVA (**c**, **d**), followed by the Holm–Sidak’s multiple comparisons test.

As PlGF plays an important role in placental development and angiogenesis by stimulating trophoblast invasion and angiogenesis via the activation of vascular endothelial growth factor (VEGF) receptor 1 (VEGFR1)^7,26,27^, we examined the role of the miR-214-3p/PlGF axis in trophoblasts and endothelial cells using conditioned medium (CM), which was harvested from HTR-8/SVneo cells exposed to normoxia (CM-N), hypoxia (CM-H), or hypoxia and an miR-214-3p inhibitor (CM-H/I) (Fig. 3a). CM was preincubated with an anti-VEGF neutralizing antibody to block VEGF activity and was used to determine the biological activity of trophoblasts and HUVECs (Fig. 3a). Treatment of trophoblasts with CM-H inhibited VEGFR1 phosphorylation and cell migration and invasion compared with treatment with CM-N; however, CM-H/I maintained the migration and invasion of trophoblasts via VEGFR1 phosphorylation, and these activities were lost following preincubation of CM-H/I with a neutralizing PlGF antibody. Notably, none of the CMs affected the functions of *Vegfr1*–knockdown cells (Fig. 3b–d and Supplementary Fig. 5a–c). Similarly, compared with CM-N, CM-H inhibited angiogenic signaling and activity, such as VEGFR1 phosphorylation, cell migration, and tube formation, in HUVECs, whereas CM-H/I maintained angiogenic activity, which was blocked by preincubation with the PlGF antibody (Fig. 3e, f and Supplementary Fig. 5d). These results suggest that HIF-1/Twist1-responsive miR-214-3p inhibits PlGF expression and secretion in trophoblasts, impairing trophoblast and endothelial cell functions via autocrine and paracrine modes of action, respectively.

### TNF-α downregulates eNOS expression via NF-κB-dependent miR-214-3p biogenesis

**Fig. 4:**
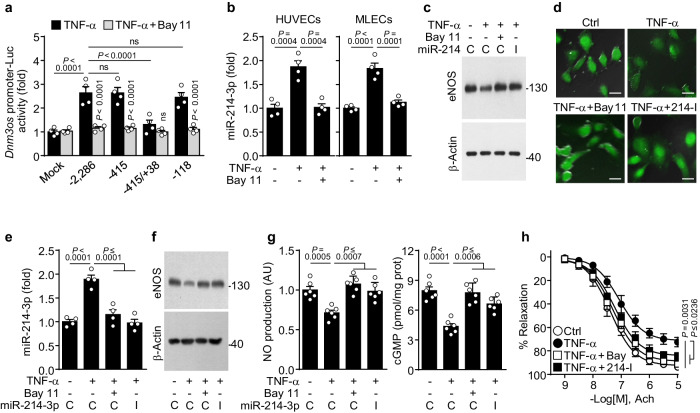
TNF-α downregulates eNOS expression via NF-κB-dependent miR-214-3p biogenesis. **a** HUVECs were transfected with the human pGL3-*Dnm3os* promoter constructs and treated with TNF-α or Bay 11-7082 (Bay 11). Luciferase activity was determined using an enzyme assay kit (*n* = 4). **b** HUVECs or MLECs were treated with TNF-α or Bay 11, and miR-214-3p was quantified via qRT‒PCR (*n* = 4). **c**, **d** MLECs were transfected with control miRNA (C) or a miR-214-3p inhibitor (I) and then treated with TNF-α or Bay 11. The levels of mouse eNOS (**c**) and NO production (green fluorescence, **d**) were determined using western blotting and DAF-FM-based fluorescence microscopy, respectively. Scale bar = 10 μm. **e**–**g** Mouse aortic vessels were transfected with control miRNA (C) or a mmu-miR-214-3p inhibitor (I), followed by treatment with TNF-α or/and Bay 11. The levels of miR-214-3p (*n* = 4, **e**), eNOS (**f**), and NO and cGMP production (*n* = 6, **g**) were determined using qRT‒PCR, western blotting, DAF-FM-based fluorescence microscopy, and ELISA. **h** Mouse aortic rings were transfected with control miRNA or a miR-214-3p inhibitor (214-I), followed by treatment with TNF-α or/and Bay 11-7082 (Bay). The vasorelaxation responses of aortic vessels to Ach were determined using myography (*n* = 4). The data are presented as the mean ± SEM. Statistical significance was determined using multiple *t* tests (**h**) and one-way (**b**, **e**, **g**) or two-way ANOVA (**a**), followed by the Holm–Sidak’s multiple comparisons test. ns not significant.

As placental hypoxia increases the levels of maternal circulating proinflammatory cytokines, including TNF-α^1,10,13^, which may contribute to eNOS downregulation and vascular dysfunction by elevating miR-214-3p and miR-155-5p levels^13–15^, we examined whether the TNF-α/NF-κB signaling pathway regulates the expression of these miRNAs in endothelial cells. Bioinformatics analysis revealed that the human (~2.3 kbp) and mouse (~1.9 kbp) *Dnm3os* promoters contain four putative NF-κB-binding motifs (Supplementary Fig. 3g–i). TNF-α treatment increased the transcriptional activity of the human *Dnm3os* promoter in HUVECs, and this activity was blocked by treatment with the NF-κB inhibitor Bay 11-7082 (Fig. 4a). Moreover, the proximal region (−118 bp) of the human *Dnm3os* promoter, which contains three putative NF-κB-binding sites, was potentially responsive to TNF-α (Fig. 4a). TNF-α treatment increased miR-214-3p biogenesis in HUVECs and mouse lung endothelial cells (MLECs), and these increases were blocked by treatment with Bay 11-7082 (Fig. 4b). MiR-155-5p expression was also upregulated in TNF-α-treated HUVECs and MLECs in an NF-κB-dependent manner (Supplementary Fig. 6a). We next examined the roles of miR-214-3p and miR-155-5p in regulating eNOS expression and vascular function. Computational prediction revealed that miR-214-3p and miR-155-5p stringently target the 3′-UTRs of mouse *eNOS* mRNA (7mer-m8) and human *eNOS* mRNA (7mer-m8), respectively (Supplementary Fig. 6b). TNF-α treatment inhibited human *eNOS* mRNA 3′-UTR activity in HUVECs and mouse *eNOS* mRNA 3′-UTR activity in MLECs in an NF-κB-dependent manner, and these effects were reversed by treatment with miR-155-5p and miR-214-3p inhibitors, respectively (Supplementary Fig. 6c, d). Transfection of human and mouse miR-155-5p and miR-214-3p mimics inhibited eNOS expression in HUVECs and MLECs in a species-specific manner (Supplementary Fig. 6e, f). Furthermore, TNF-α treatment decreased eNOS expression, NO production, and cGMP synthesis in MLECs, and these changes were all reversed by treatment with Bay 11-7082 or a miR-214-3p inhibitor (Fig. 4c, d and Supplementary Fig. 6g, h). Moreover, treatment of mouse aortic vessels with TNF-α increased miR-214-3p biogenesis and decreased eNOS expression, NO/cGMP production, and acetylcholine (Ach)-mediated vasorelaxation compared with those of the untreated control vessels; however, these effects were blocked by treatment with Bay 11-7082 or a miR-214-3p inhibitor (Fig. 4e–h). These results suggest that TNF-α impairs eNOS-dependent vascular function by increasing the NF-κB-dependent biogenesis of mouse miR-214-3p and human miR-155-5p.

### MiR-214-3p administration causes PE-like symptoms

**Fig. 5:**
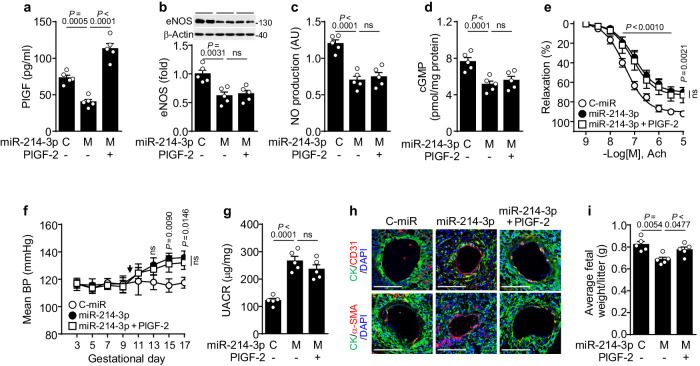
Synthetic miR-214-3p administration induces PE-like phenotypes in pregnant mice. Pregnant mice were infused with locked nucleic acid-based control miRNA (C), synthetic miR-214-3p mimics (M), or miR-214-3p plus rmPlGF-2 (PlGF-2) using an osmotic pump from GD 9.5 to GD 17. **a** Circulating PlGF levels were determined using an ELISA kit (*n* = 5). **b** Levels of eNOS were determined in maternal aortic vessels using western blotting and quantified using ImageJ (*n* = 5). **c**, **d** The levels of NO and cGMP synthesis were determined in aortic vessels and their lysates using DAF-FM-based fluorescence microscopy and an ELISA kit, respectively (*n* = 5). **e** Vasorelaxation responses of aortic vessels to Ach were determined using myography (*n* = 5). **f** The mean blood pressure (BP) was determined using a noninvasive tail-cuff method (*n* = 5). The arrow indicates surgical implantation of the osmotic pump. **g** The urine albumin-to-creatinine ratio (UACR) was calculated after determining the levels of these molecules using ELISA kits (*n* = 5). **h** Representative images of placental tissues stained with DAPI (nuclear marker) and antibodies against cytokeratin (CK, a trophoblast marker), CD31 (an endothelial cell marker), or α‐smooth muscle actin (α-SMA, a smooth muscle cell marker) and visualized using confocal microscopy. Scale bar = 100 μm. **i** Average fetal weight at GD 17 (5–10 fetuses/litter, *n* = 5 litters). The data are presented as the mean ± SEM. Statistical significance was determined using multiple *t* tests (**e**, **f**) or one-way ANOVA (**a**–**d**, **g**, **i**), followed by the Holm–Sidak’s multiple comparisons test. ns not significant.

To determine whether miR-214-3p expression is a risk factor for PE, we continuously infused pregnant mice with control miRNA, miR-214-3p mimics, or miR-214-3p plus recombinant mouse PlGF-2 (rmPlGF-2) using an osmotic pump from gestational day (GD) 9.5 to GD 17, and the development of PE-like characteristics and symptoms was determined. Compared with the control miRNA infusion, the miR-214-3p mimic administration decreased PlGF levels but not sFlt-1 levels in the circulation of pregnant mice, increasing the serum sFlt-1/PlGF ratio, which is known as a diagnostic criterion for PE (Fig. 5a and Supplementary Fig. 7a). In addition, miR-214-3p mimic administration significantly reduced eNOS expression, NO production, cGMP synthesis, and Ach-induced vasorelaxation in the aortic vessels of pregnant mice (Fig. 5b–e), elevating the mean blood pressure without altering the heart rate (Fig. 5f and Supplementary Fig. 7b). MiR-214-3p mimic administration also increased the urine albumin-to-creatinine ratio (UACR), an indicator of proteinuria, in pregnant mice and caused renal glomerular injuries histologically characterized by glomerular enlargement, glomerular endotheliosis, and decreased Bowman′s space area (Fig. 5g and Supplementary Fig. 7c–e). Since fetal growth restriction is one of the most common complications of PE and arises primarily from inappropriate trophoblast invasion, poor uteroplacental vessel development, and defective spiral artery remodeling^1,7^, the role of exogenous miR-214-3p in spiral artery remodeling was investigated in a hypoxic pregnant mouse model. Immunohistochemical analysis revealed that miR-214-3p infusion inhibited maternal spiral artery remodeling, with a reduced arterial diameter and decreased replacement of CD31^+^ endothelial cells and α‐smooth muscle actin (α-SMA)^+^ vascular smooth muscle cells in spiral arteries by cytokeratin (CK)^+^ trophoblasts (Fig. 5h and Supplementary Fig. 7f). As expected, miR-214-3p infusion significantly reduced fetal body weight (Fig. 5i and Supplementary Fig. 7g). However, continuous rmPlGF-2 administration reversed only the inhibitory effects of miR-214-3p on spiral artery remodeling and fetal growth but not the effects on other PE-like symptoms (Fig. 5a–i and Supplementary Fig. 7a–g). These results suggest that miR-214-3p causes PE-like symptoms in pregnant mice by downregulating PlGF and inhibiting the eNOS/NO/cGMP axis, which play important roles in placental arterial remodeling/fetal growth and maternal vascular function/glomerular endothelial function, respectively.

### MiR-214-3p deficiency maintains PlGF and eNOS levels in an experimental mouse model of PE

**Fig. 6:**
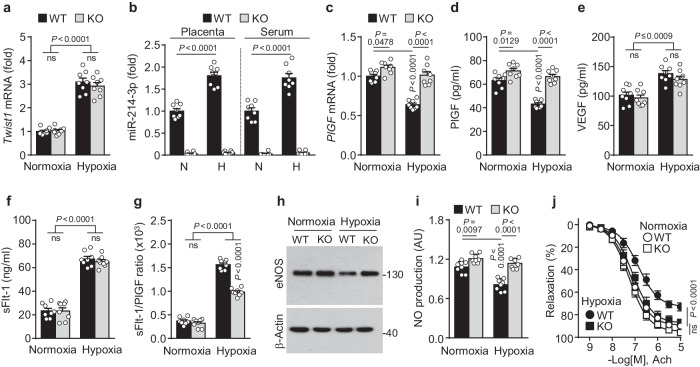
MiR-214-3p deficiency prevents PE-like characteristics in hypoxic pregnant mice. Pregnant WT and miR-214-3p KO mice were exposed to normoxia (N) or hypoxia (H) from GD 9.5 to GD 17.5. **a** *Twist1* mRNA levels were determined in placental tissues using qRT‒PCR (*n* = 8). **b** MiR-214-3p levels were determined in placental tissues and maternal sera using qRT‒PCR (*n* = 8). **c** *PlGF* mRNA levels were determined in placental tissues using qRT‒PCR (*n* = 8). **d**–**f** Serum levels of PlGF, VEGF, and sFlt-1 were quantified using ELISA kits (*n* = 8). **g** The sFlt-1/PlGF ratio was calculated from the serum concentration of each protein (*n* = 8). **h**, **i** Levels of eNOS and NO were determined in mouse aortic vessels using western blotting and DAF-FM-based fluorescence microscopy, respectively (*n* = 8). **j** The vasorelaxation responses of mouse aortic rings to Ach were determined using myography (*n* = 5). The data are presented as the mean ± SEM. Statistical significance was determined using multiple *t* tests (**j**) or one-way ANOVA (**a**–**g**, **i**), followed by the Holm–Sidak’s multiple comparisons test. ns, not significant.

We further investigated the role of miR-214-3p in the pathogenesis of PE using miR-214-3p knockout (KO) mice, which were generated using CRISPR/Cas9-mediated deletion of 14 bp of miR-214-3p located in intron 14 of *Dnm3os* (Supplementary Fig. 8a–c). MiR-214-3p-KO mice showed no differences in placental and hepatic Dnm3 protein levels, morphological appearance, and body weight (Supplementary Fig. 8d–f). To develop a gestational hypoxia-induced PE mouse model^28^, we exposed pregnant WT and KO mice to hypoxia (9.5% O_2_), and the serum biomarker levels of PE were analyzed. Hypoxic insult increased *Twist1* mRNA levels in the placentas of the WT and miR-214-3p KO mice, but there was no difference in *Twist1* expression between the mice with different genotypes (Fig. 6a). Hypoxia also elevated miR-214-3p levels in the placental tissues and maternal sera of the WT mice compared with those observed under normoxia, whereas miR-214-3p was not detectable in the miR-214-3p KO mice under either condition (Fig. 6b). As expected, compared with normoxic conditions, hypoxic conditions significantly reduced *PlGF* mRNA and protein levels in the placentas and sera of pregnant WT mice but not in those of miR-214-3p KO mice. Additionally, PlGF expression levels under normoxic conditions were greater in the miR-214-3p KO mice than in the WT mice (Fig. 6c, d). We also examined the expression levels of miR-214-4p and PlGF in primary trophoblasts isolated from pregnant WT and miR-214-3p KO mice exposed to normoxia or hypoxia. Similar to that in placental tissues, miR-214-3p was markedly upregulated in primary trophoblasts from the hypoxia-exposed WT mice but was not detectable in those from the miR-214-3p KO mice; however, under hypoxia, *PlGF* mRNA levels were significantly reduced in placental trophoblasts of the WT mice and increased in those of the KO mice (Supplementary Fig. 9a, b). The serum levels of other pro- and antiangiogenic factors, such as VEGF, sFlt-1, and sEng, were increased in both pregnant WT and miR-214-3p KO mice under hypoxic conditions but did not differ between the mouse genotypes under the same oxygen conditions (Fig. 6e, f and Supplementary Fig. 9c). Under hypoxic conditions, the sFlt-1/PlGF ratio was greater in the WT mice than in the miR-214-3p KO mice (Fig. 6g). Similarly, the serum levels of TNF-α, IL-1β, and IL-6 were increased in both the pregnant WT and miR-214-3p KO mice under hypoxic conditions but showed no differences between mouse genotypes under the same oxygen conditions (Supplementary Fig. 9d). Since inflammatory cytokines upregulate endothelial miR-214-3p, which targets eNOS mRNA^13–15^ (Fig. 4), the effects of hypoxia on miR-214-3p and eNOS expression were examined. Compared with normoxia, hypoxia significantly increased miR-214-3p expression in aortic tissues from WT but not miR-214-3p mice (Supplementary Fig. 9e). Hypoxia significantly decreased eNOS expression and NO synthesis in the maternal aortas of the WT mice but not in those of the miR-214-3p KO mice, while the aortic eNOS protein and NO levels were greater in the miR-214-3p KO mice than in the WT mice under normoxic conditions (Fig. 6h, i and Supplementary Fig. 9f, g). Similar results were observed for the expression of miR-214-3p and eNOS in mouse aortic endothelial cells (Supplementary Fig. 9h, i). Moreover, the relaxation responses to Ach were significantly lower in the aortic vessels of the hypoxic pregnant WT mice than in those of the hypoxic pregnant miR-214-3p KO mice (Fig. 6j). These results indicate that miR-214-3p deletion improves the serum sFlt-1/PlGF ratio and vascular reactivity in hypoxia-exposed pregnant mice by restoring PlGF and eNOS expression.

### MiR-214-3p KO ameliorates hypoxia-induced PE-like symptoms in pregnant mice

**Fig. 7:**
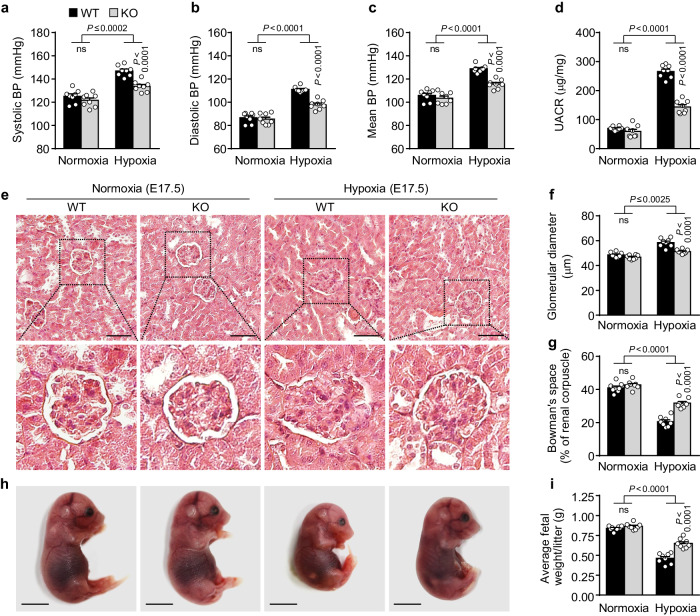
MiR-214-3p deficiency ameliorates PE-like symptoms in hypoxic pregnant mice. Pregnant WT and miR-214-3p KO mice were treated as described in Fig. 6. Average values of systolic (**a**), diastolic (**b**), and mean arterial blood pressure (BP) (**c**) were determined using a noninvasive tail-cuff method (*n* = 8). **d** The UACR was calculated after determining urinary albumin and creatinine levels using albumin ELISA kits (*n* = 8). **e** Representative histological images of maternal renal tissues stained with H&E. Scale bar = 50 μm. **f** The glomerular diameter was calculated using ImageJ and is expressed as the average value of five random glomeruli in each renal tissue section (*n* = 8). **g** Bowman′s capsule space was calculated by subtracting the glomerular tuft area from the renal corpuscle area (*n* = 8). **h** Representative photographs of GD 17.5 fetuses from WT and miR-214-3p KO mice (6–11 fetuses/litter, *n* = 8 litters). Scale bar = 0.5 cm. **i** Average fetal weight at GD 17.5 (*n* = 8 litters). The data are presented as the mean ± SEM. Statistical significance was determined using one-way ANOVA, followed by the Holm–Sidak’s multiple comparisons test. ns not significant.

Next, we evaluated the pathological symptoms and characteristics of PE in hypoxic pregnant WT and miR-214-3p KO mice. Compared with normoxic conditions, hypoxic treatment increased the systolic, diastolic, and mean arterial blood pressure in pregnant WT mice, all of which were significantly reduced in pregnant miR-214-3p KO mice; however, the blood pressure did not differ between the WT and miR-214-3p KO mice under normoxic conditions (Fig. 7a–c). The UACR was markedly increased in the hypoxia-exposed pregnant WT and miR-214-3p KO mice but was significantly lower in the latter, whereas no difference was observed in the basal UACR between the WT and miR-214-3p KO mice under normoxia (Fig. 7d). Histological analysis of maternal kidneys revealed that the extent of hypoxia-induced renal glomerular injuries, such as glomerular enlargement, glomerular endotheliosis, and decreased Bowman′s space area, was lower in the pregnant miR-214-3p KO mice than in the WT mice, while a normal kidney architecture was observed in both WT and miR-214-3p KO mice under normoxia (Fig. 7e–g). In addition, the fetal and placental weights were significantly decreased in the WT and miR-214-3p KO mice exposed to hypoxia but to a much lesser extent in the miR-214-3p KO mice, whereas no differences were observed in these weights between the two mouse genotypes under normoxic conditions (Fig. 7h, i and Supplementary Fig. 10a, b). These results suggest that miR-214-3p deletion ameliorates PE-like symptoms in hypoxic pregnant mice.

### MiR-214-3p deficiency improves spiral artery remodeling

**Fig. 8:**
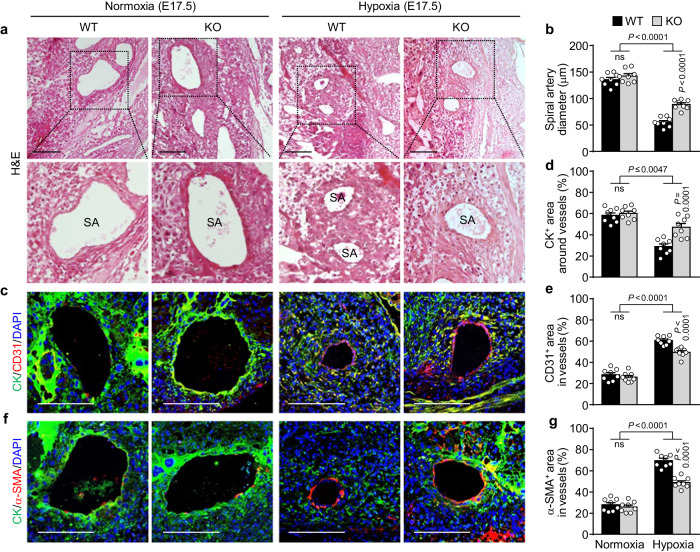
MiR-214-3p deficiency alleviates spiral artery remodeling in hypoxic pregnant mice. Pregnant WT and miR-214-3p KO mice were treated as described in Fig. 6. **a** Representative images of placental tissues stained with H&E. Scale bar = 100 μm. SA spiral artery. **b** The diameter of the spiral arteries was measured using ImageJ and is expressed as the average value of five random vessels in each placental tissue section (*n* = 8 litters). **c**, **f** Representative images of placental tissues stained with DAPI (nuclear marker) and antibodies against cytokeratin (CK, a trophoblast marker), CD31 (an endothelial cell marker), or α‐smooth muscle actin (α-SMA, a smooth muscle cell marker) and visualized using confocal microscopy. Scale bar = 100 μm. Areas of CK^+^ trophoblasts (**d**), CD31^+^ endothelial cells (**e**), and α-SMA^+^ smooth muscle cells (**g**) around or within vessels were determined using ZEN software and are expressed as the mean value of five random vessels in each placental tissue section (*n* = 8 litters). The data are presented as the mean ± SEM. Statistical significance was determined using one-way ANOVA, followed by the Holm–Sidak’s multiple comparisons test. ns not significant.

Subsequently, the role of miR-214-3p in spiral artery remodeling was investigated in a hypoxic pregnant mouse model. Macroscopic analysis revealed that hypoxia reduced the density and diameter of the decidual spiral arteries more significantly in pregnant WT mice than in miR-214-3p KO mice, whereas normoxic conditions did not alter the arterial density or diameter between pregnant WT and miR-214-3p KO mice (Supplementary Fig. 10c–e). In addition, hematoxylin and eosin (H&E) staining confirmed that hypoxia decreased the luminal diameter of spiral arteries in both pregnant WT and miR-214-3p KO mice but to a much lesser extent in the latter (Fig. 8a, b), suggesting that miR-214-3p plays an important role in impaired spiral artery remodeling. Further examination via immunohistochemical analysis revealed that under hypoxic conditions, spiral artery remodeling was impaired in both pregnant WT and miR-214-3p KO mice but to a much lesser extent in the latter, as confirmed by the greater invasion of CK^+^ trophoblasts and lower coverage of CD31^+^ endothelial cells and α-SMA^+^ vascular smooth muscle cells in the spiral arteries of the miR-214-3p KO mice than in those of the WT mice. However, normoxic conditions did not alter the levels of these spiral artery remodeling markers between pregnant mice of the two genotypes (Fig. 8c–g). Thus, miR-214-3p deficiency significantly increased the ratio of trophoblasts to endothelial cells or vascular smooth muscle cells in the spiral arteries of hypoxic pregnant mice (Supplementary Fig. 11a, b). These results suggest that hypoxia- and/or inflammation-induced miR-214-3p expression is an important risk factor for impaired decidual spiral artery remodeling.

## Discussion

Accumulating evidence suggests that hypoxia and inflammation play important roles in the pathogenesis of PE; however, the underlying molecular mechanisms are still unclear. Here, we investigated the potential roles of HIF-1/Twist1- and NF-κB-responsive miR-214-3p in the pathogenesis of PE. Our results demonstrated that hypoxia and TNF-α increased miR-214-3p expression, which inhibited PlGF and eNOS expression in trophoblasts and endothelial cells, eventually impairing trophoblast invasion and vascular function. Continuous administration of synthetic miR-214-3p downregulated PlGF and eNOS expression in pregnant mice and caused PE-like symptoms, including hypertension and proteinuria. We also found that miR-214-3p deletion prevented a decrease in circulating PlGF levels and vascular eNOS expression in hypoxic pregnant mice, thereby alleviating glomerular injury-mediated proteinuria and endothelial dysfunction-induced hypertension. Moreover, miR-214-3p deficiency improved uterine spiral artery remodeling through effective replacement of endothelial and vascular smooth muscle cells by invasive endovascular trophoblasts, thereby promoting fetal growth. These results suggest that hypoxia- and inflammation-induced miR-214-3p expression contributes to the pathogenesis of PE and its complications by downregulating placental PlGF and vascular eNOS expression.

During early pregnancy, abnormal placental implantation elicits uteroplacental hypoxia, which decreases proangiogenic PlGF expression and elevates antiangiogenic sFlt-1 and sEng production through stabilization of the HIF-1α protein, consequently leading to trophoblast and endothelial dysfunction, spiral artery abnormalities, and glomerular injury^1^. Previous studies have shown that hypoxic insult^28^, trophoblast-specific HIF-1α expression^29^, or exogenous sFlt-1, a PlGF antagonist^30,31^, induces PE-like symptoms in murine models, emphasizing that hypoxia/HIF-1-dependent downregulation of PlGF expression contributes to the pathophysiology of PE. This finding indicates that the hypoxic pregnant mice used in this study are an appropriate model for investigating the pathogenic mechanism of PE^28^, although other animal models have also been reported^32,33^. Indeed, we found that hypoxic pregnant mice exhibited PE-like characteristics, including an increased sFlt-1/PlGF ratio, glomerular injury, impaired spiral artery remodeling, hypertension, proteinuria, and fetal growth restriction. Accordingly, direct administration of recombinant PlGF alleviated hypertension and plasma sFlt-1 levels in a pregnant rat model of PE^34^. Contrary to clinical findings showing that reduced PlGF levels are a major risk factor for PE, genetic deletion of *PlGF* has been shown to ameliorate the PE-like phenotype in pregnant catechol-O-methyltransferase-deficient mice, a spontaneous mouse model of PE^35^. These results highlight the complexity of the pathogenesis of PE owing to its multiple risk factors, such as imbalanced pro- and antiangiogenic factors (e.g., PlGF, sFlt-1, and sEng) levels, immune activation, reduced eNOS/NO bioactivity, and oxidative stress^1,13^.

PlGF is highly expressed in placental villous trophoblasts under normal physiological conditions, facilitating trophoblast growth and differentiation, angiogenesis, and spiral artery remodeling^9,27^. Thus, circulating PlGF concentrations are significantly lower in women with preterm PE than in healthy pregnant women, and these reduced PlGF levels precede the clinical onset of the disease^36,37^. PlGF expression is suppressed in trophoblasts under hypoxic conditions induced by shallow placentation; however, the expression is upregulated by hypoxia or overexpressed HIF-1α in nontrophoblast cells, such as endothelial cells, cardiac fibroblasts, and some tumor cells^5,6,8,9,38^. This finding suggests that the hypoxia/HIF-1 axis regulates PlGF expression in a cell type-specific manner^9^, although the underlying mechanism remains unclear. Our data showed that hypoxia increased the transcription of *PlGF* in both trophoblasts and nontrophoblasts but effectively decreased its mRNA and protein levels only in trophoblasts; these effects were prevented by treatment with a HIF-1α inhibitor, indicating that the hypoxia/HIF-1 axis negatively regulates PlGF expression at the post-transcriptional level in trophoblasts. This regulation may be due to the cell type-specific expression of HIF-1-responsive factors, including miRNAs, which mediate post-transcriptional gene silencing. In fact, specific knockdown of *Dicer*, whose product RNase III is essential for the miRNA biogenesis pathway^23^, potentially rescued hypoxia-induced downregulation of PlGF expression, but this activity did not reach the level of its promoter activity in trophoblasts. These results suggest that hypoxia downregulates PlGF expression in both miRNA-dependent and miRNA-independent manners in trophoblasts. Notably, our results showed that HIF-1-responsive miR-214-3p expression was upregulated in trophoblasts but not in HUVECs or HASMCs. These findings suggest that miR-214-3p, whose expression is upregulated in placental trophoblasts by hypoxia, contributes to the pathogenesis of PE through post-transcriptional repression of *PlGF* expression. However, the mechanisms by which miR-214-3p levels are differentially regulated in trophoblasts and nontrophoblasts are still unknown. These mechanisms may involve cell type-specific regulatory factors for miRNA biogenesis, including superenhancer activity, chromatin accessibility, nuclear export of pre-miRNA, and miRNA-processing enzymes^39–43^. Therefore, further studies are required to investigate the molecular mechanisms underlying cell type-specific miR-214-3p expression under hypoxic conditions. Conversely, the results also suggest that hypoxia negatively regulates trophoblastic PlGF expression in miRNA-dependent and miRNA-independent manners. The miRNA-independent mechanism may be linked to hypoxia-induced ER stress, which is associated with IRE1- and PERK-dependent post-transcriptional repression (i.e., mRNA decay and translational inhibition) of some genes, including *PlGF*^44,45^. In addition, the possibility that hypoxia-induced REDD1 may repress the translation of PlGF mRNA cannot be excluded^46^.

The transcription factor HIF-1 regulates the expression of angiogenic, metabolic, and survival-related genes as well as various miRNAs by binding to HREs in their promoters^47,48^. Notably, we found that the *Dnm3os* promoter does not contain an HRE consensus sequence but contains several E-box motifs that are recognized by the transcription factor Twist1. HIF-1 promotes Twist1 expression by binding to the HRE in the proximal region (−83/−79) of its promoter^24^, suggesting that miR-214-3p biogenesis is mediated by HIF-1-dependent Twist1 expression. Similarly, we found that hypoxia-induced suppression of PlGF expression in trophoblasts was rescued by *Twist1* knockdown, suggesting that hypoxia inhibits PlGF expression by promoting HIF-1/Twist1-mediated miR-214-3p biogenesis, consistent with the results of previous studies showing an important role of Twist in miR-199a/214 cluster expression^19,25^. Collectively, these results suggest that miR-214-3p biogenesis impairs trophoblast invasion and spiral artery remodeling by suppressing PlGF expression in hypoxic placentas.

In addition to downregulation of PlGF expression, dysregulation of the eNOS/NO axis is considered a cause of vascular dysfunction and hypertension in PE^31,49^. Pharmacological inhibition of NOS activity or genetic deletion of *eNOS* induces PE-like characteristics, including hypertension, proteinuria, impaired spiral artery remodeling, and fetal growth restriction, in murine models^49–52^. Several studies have shown decreased NO production or bioavailability in women with PE but not in all patients^13,53^. This finding may be attributed to potential eNOS downregulation, despite increased expression of inducible NOS, under chronic inflammatory conditions associated with the pathogenesis of PE^10,13^. Therefore, the suppression of the eNOS/NO axis plays an important role in the induction of PE-associated vascular dysfunction during pregnancy^54^. In fact, placental hypoxia results in NF-κB activation and increased circulating TNF-α and IL-1β levels, which impair trophoblast and endothelial cell functions in a miRNA-dependent manner^10–16,19,55^; thus, PE can be considered a type of inflammatory disease^56^. This finding suggests that NF-κB activation induces a PE-like phenotype via the suppression of PlGF and eNOS expression under hypoxic and systemic inflammatory conditions associated with early-onset PE. Although eNOS is constitutively expressed in endothelial cells, its expression is regulated by several miRNAs, including miR-31-5p, miR-155-5p, miR-214-3p, and miR-584-5p, whose expression is upregulated in response to inflammation as well as in the circulation of patients with PE^13–15,57–59^. However, the downregulation of eNOS expression by these miRNAs shows a species-specific pattern, such as miR-31-5p, miR-155-5p, and miR-584-5p against human *eNOS* mRNA and miR-214-3p against mouse *eNOS* mRNA^13–15,58^. Given our results showing that miR-214-3p targets the 3′-UTRs of mouse *eNOS* mRNA and mouse and human *PlGF* mRNA, this miRNA is a useful target for investigating the role of eNOS and PlGF in the pathogenesis of PE using mouse models. We also found that miR-214-3p deletion ameliorated PE-like symptoms by maintaining eNOS and PlGF levels in hypoxic pregnant mice. However, NF-κB-responsive miR-31-5p and miR-155-5p expression may be clinical risk factors for PE, as these molecules suppress the eNOS/NO axis during human pregnancy^13–15^.

As PE is a complex disease that naturally occurs only in humans and gorillas^60^, standard animal models of this disease are not well established. Although some animal models have been developed using oral administration of an NOS inhibitor, eNOS deficiency, infusion of sFlt-1 or TNF-α, and gene-targeting approaches^30–33,61^, pregnant mice exposed to hypoxia are generally accepted as simple animal models of PE^28^, as hypoxia is considered a major cause of PE and activates the inflammatory pathway^1,56^, probably via hypoxia-induced upregulation of REDD1, an atypical NF-κB activator^46,62,63^. Accordingly, we found that controlled hypoxia-induced PE-like characteristics and symptoms were induced in pregnant WT mice through reductions in serum PlGF levels and eNOS expression; however, these effects were not observed in pregnant miR-214-3p KO mice. Notably, chronic administration of synthetic miR-214-3p resulted in the development of PE-like features in pregnant mice, suggesting that this protocol can be used to construct a new experimental mouse model for investigating the pathogenesis of PE. Based on our data and previous results^13,19^, the HIF-1/Twist1/miR-214-3p axis in the hypoxic placenta may be responsible for impaired trophoblast invasion, inadequate spiral artery remodeling, and fetal growth restriction through the downregulation of PlGF expression during pregnancy. In addition, the inflammatory NF-κB/miR-214-3p axis causes hypertension and proteinuria by inhibiting eNOS expression in maternal endothelial cells of hypoxic pregnant mice. We found no significant differences in the circulating levels of the inflammatory cytokines VEGF, sFlt-1, and sEng between hypoxic pregnant WT and miR-214-3p KO mice. These phenomena are likely due to the global exposure of pregnant mice to chronic hypoxia, which induces persistent inflammation^64^, and strongly suggest that miR-214-3p-mediated downregulation of PlGF and eNOS expression is crucial for the pathogenesis of PE.

In conclusion, we demonstrated that placental hypoxia and systemic inflammation inhibited PlGF expression in trophoblasts and eNOS expression in endothelial cells mainly via the HIF-1/Twist1/miR-214-3p and NF-κB/miR-214-3p axes, respectively, causing trophoblast and endothelial cell dysfunction. Chronic administration of synthetic miR-214-3p downregulated PlGF and eNOS expression in pregnant mice, resulting in a PE-like phenotype; however, miR-214-3p deficiency protected against PE-like symptoms and characteristics in hypoxic pregnant mice by maintaining maternal circulating PlGF and vascular eNOS levels. Taken together, our findings indicate that miR-214-3p plays a crucial role in the development of PE-like symptoms in mice by downregulating PlGF expression in trophoblasts and eNOS expression in endothelial cells (Supplementary Fig. 12), highlighting miR-214-3p and its biogenesis pathways as potentially useful therapeutic targets for the treatment or prevention of PE.

### Supplementary information

Supplementary Information

## Data Availability

Correspondence and requests for materials should be addressed to Y.M.K.
