# Publisher Correction: Alleviation of preeclampsia-like symptoms through PlGF and eNOS regulation by hypoxia- and NF-κB-responsive miR-214-3p deletion

**DOI:** 10.1038/s12276-024-01275-2

**Published:** 2024-07-18

**Authors:** Suji Kim, Sungbo Shim, Jisoo Kwon, Sungwoo Ryoo, Junyoung Byeon, Jungwoo Hong, Jeong-Hyung Lee, Young-Guen Kwon, Ji-Yoon Kim, Young-Myeong Kim

**Affiliations:** 1 Department of Molecular and Cellular Biochemistry, School of Medicine, Kangwon National University, Chuncheon, 24341 Republic of Korea; 2 Department of Biochemistry, Chungbuk National University, Cheongju, 28644 Republic of Korea; 3 Department of Anesthesiology and Pain Medicine, Hanyang University Hospital, Hanyang University College of Medicine, Seoul, 04763 Republic of Korea; 4 Department of Biological Sciences, Kangwon National University, Chuncheon, 24341 Republic of Korea; 5 Department of Biochemistry, College of Natural Sciences, Kangwon National University, Chuncheon, 24341 Republic of Korea; 6 Advanced Institute of Technology, Curacle Co. Ltd, Seoul, 06694 Republic of Korea

**Keywords:** Mechanisms of disease, Embryonic induction, Pre-eclampsia, Predictive markers

Correction to: *Experimental & Molecular Medicine* 10.1038/s12276-024-01237-8, published online 09 May 2024

In this article the publication date of 9 May 2024 was incorrect and should have been 3 June 2024. The original article has been corrected.

