# Supplementary material for: Alleviation of preeclampsia-like symptoms through PlGF and eNOS regulation by hypoxia- and NF-κB-responsive miR-214-3p deletion
Source: Exp Mol Med. 2024 Jun 3;56(6):1388–400. doi: 10.1038/s12276-024-01237-8 (PMC11263402; doi:10.1038/s12276-024-01237-8)
Supplement: Supplementary file 1 — Supplementary Information [file 12276_2024_1237_MOESM1_ESM.pdf]

SUPPLEMENTARY INFORMATION

Alleviation of preeclampsia-like symptoms through PlGF and eNOS regulation by hypoxia- and NF-κB-responsive miR-214-3p deletion

Materials

Cell culture media and supplements, Lipofectamine RNAiMAX (13778150), and Lipofectamine 3000 (L3000015) were purchased from Invitrogen Life Technologies (Carlsbad, CA, USA). SiRNAs for control (sc-37007), *Dicer* (sc-40489), *HIF1A* (sc-35561), *Twist1* (sc-38604), and *Vegfr1* (sc-29319) were purchased from Santa Cruz Biotechnology (Santa Cruz, CA, USA). Hsa-miR-155-5p mimic (MSY0000646), mmu-miR-155-5p mimic (MSY0000165), hsa-miR-214-3p mimic (MSY0000271), mmu-miR-214-3p mimic (MSY0000661), control miRNA (1027281), hsa-miR-214-3p inhibitor (MIN0000271), mmu-miR-214-3p inhibitor (MIN0000661), and miRNA inhibitor control (1027271) were purchased from QIAGEN (Hilden, Germany). Antibodies against PlGF (sc-518003; 1:1,000) and Twist1 (sc-81417; 1:1,000) were purchased from Santa Cruz Biotechnology. Antibodies against HIF-1α (MAB1536; 1:1,000), phosphorylated VEGFR-1 (AF4170; 1:1,000), human VEGF (neutralizing, MAB293), and human PlGF (neutralizing, MAB11197), as well as recombinant human TNF-α (210-TA) and mouse TNF-α (410-MT), were purchased from R&D Systems (Minneapolis, MN, USA). The antibody against VEGFR-1 (2893; 1:1,000) was purchased from Cell Signaling Technology (Danvers, MA, USA). Antibodies against eNOS (610297; 1:1,000), Dnm3 (14737-1-AP; 1:1,000), and β-actin (A5441; 1:5,000) were purchased from BD Biosciences (Franklin Lakes, NJ, USA), Proteintech (Rosemont, IL, USA), and Sigma-Aldrich (St. Louis, MO, USA), respectively. 4-Amino-5methylamino-2,7-difluorofluorescein (DAF-FM) diacetate was obtained from Molecular Probes (Eugene, OR, USA). IDF-11774 (HY-111387) was from MedChem Express (Monmouth Junction, NJ, USA). Bay 11-7082 (196870) and acetylcholine (A6625) were purchased from Sigma-Aldrich.

Generation of miR-214-3p KO mice

MiR-214-3p KO mice were generated using the CRISPR/Cas9 system. Briefly, single-guide RNAs (sgRNAs; Supplementary Table 1) were cloned into the pT7-Guide-IVT vector (GE100025, Origene, Rockville, MD, USA). The constructs were linearized using the restriction enzyme BsmBI (ER0451, Thermo Fisher Scientific, Waltham, MA, USA) and used as templates for *in vitro* transcription to yield sgRNAs using the MEGAshortscript T7 kit (AM1334, Thermo Fisher Scientific). Cas9 mRNA (50 ng/μl final concentration) and sgRNAs (50 ng/μl final concentration) were mixed in TE buffer (10 mM Tris, 0.1 mM EDTA, pH 7.5) and injected into the cytoplasm of C57BL/6 zygotes using micromanipulators. The zygotes were then transferred into the uteri of pseudopregnant recipient females at 2.5 days post-coitum. After birth, DNA was extracted from the tails of the pups, and the deletion of the miR-214 precursor sequence was analyzed using PCR with its specific primers, followed by further confirmation by sequencing analysis of the amplicons. The founder lines were expanded and maintained under a 12-h light:12-h dark cycle and provided free access to a standard chow diet (JA Bio, Republic of Korea) and water in a pathogen-free animal facility. All animal experiments were approved by the Institutional Animal Care and Use Committee of the Kangwon National University (KW-200305-3)

and followed the National Institutes of Health Guide for the Care and Use of Laboratory Animals (NIH Publication, 8th edition, 2011).

Isolation of placental trophoblasts and aortic endothelial cells

Mouse primary trophoblasts were isolated using a protocol modified from previous methods[1,2]. Briefly, mouse placentas were separated from the underlying endometrium in a sterile dish containing RPMI 1640 medium using dissecting forceps and incubated in dissociation medium (serum-free RPMI 1640 supplemented with 1 mg/ml collagenase IV [C5138, Sigma-Aldrich, St. Louis, MO] and 20 μg/ml DNase I [DN25, Sigma-Aldrich]) (20 ml/g tissue) for 1 h at 37°C, with periodic pipetting to separate cells. Cells were washed to remove dissociation medium and filtered through 40-μm cell strainers. After washing, cell pellets were resuspended in 5 ml RPMI 1640, layered on the top of a preformed Percoll gradient (65 %–25) and centrifuged at 730 ×*g* at 4°C without braking for 30 min. Trophoblasts were collected from the layer between the 45% and 35% (density 1.050-1.060 g/ml) Percoll aliquots. Mouse aortic endothelial cells were isolated as described previously[3]. In brief, abdominal aorta was harvested, and its lumen was washed with serum-free medium. A 24-gauge cannula was inserted into the proximal site of the aorta and ligated with a silk thread. The other side was bound, filled with 2 mg/ml collagenase II (NC9870009, Worthington Biochemical Co. Lakewood, NJ, USA), and incubated for 40 min at 37°C. Endothelial cells were collected from aorta by flushing with 5 ml of RPMI 1640 supplemented with 20% FBS and washed by centrifugation at 1,000 ×*g* for 5 min. The precipitate was gently suspended in an appropriate volume of RPMI 1640. The trophoblasts and endothelial cells were used to analyze miRNA and mRNA expression.

MiRNA profiling and quantitative real time PCR (qRT−PCR) analysis

Expression of miRNAs in HTR-8/SVneo cells exposed to normoxia or hypoxia for 24 h was analyzed at Macrogen (Seoul, Korea) using the Affymetrix miRNA expression microarray version 3.0 (902017). Total RNA was extracted from tissues, cells, and sera using the miRNeasy Mini kit (217084, QIAGEN) or miRNeasy serum/plasma kit (217184, QIAGEN), and cDNA was then synthesized using 1 μg RNA and an miScript II RT Kit (218161, QIAGEN). Thereafter, miRNA levels were quantified via qPCR using the miScript SYBR Green PCR kit (218073, QIAGEN), miR-214-3p primers (MS00031605 for human; MS00032571 for mouse), miR-199a-3p primer (MS00007602 for human), SNORD95 miScript miRNA primer (H/M/R:MS00033726, QIAGEN), and human pre-miR-214-3p primers (Supplementary Table 1) according to methods described previously[4,5]. MiRNA levels were normalized to that of the housekeeping gene *SNORD95*. For mRNA quantification, total RNA was extracted from cultured cells and placental tissues using the TRIzol reagent (Invitrogen) and used to synthesize first strand cDNA using M-MLV Reverse Transcriptase (Promega, Madison, WI, USA), followed by quantification of *PlGF* and *eNOS* mRNA levels using qPCR and target-specific primers

(Supplementary Table 1). mRNA levels were normalized to that of the glyceraldehyde-3-phosphate dehydrogenase (*GAPDH*) gene.

In vitro endothelial cell angiogenesis and trophoblast migration/invasion assays

HUVEC migration and tube formation were assessed as described previously[5]. For the migration assay using Transwell plates with polycarbonate filters (8-μm pore size; Corning Inc.), CM were placed in the lower wells, and HUVECs (2×10^4 cells) were loaded in the upper wells, followed by incubation at 37°C for 4 h. The cells that migrated to the lower side of the filter were quantified by counting those in all fields in each assay, as described above for the trophoblast invasion assay. HUVEC tube formation was determined after treatment with CM on a layer of growth factor-reduced Matrigel in a humidified CO_2 incubator. After culture for 24 h, tube formation was observed under an Olympus IX71 microscope, and images were captured using a video graphic system. Tube formation was then quantified using ImageJ software (NIH, Bethesda, MD, USA). Trophoblast migration and invasion were assessed by scratch-wound assay and Boyden chamber assay, respectively. For the scratch-wound assay, a linear scratch was gently made at the center of the HTR-8/SVneo cell monolayer using the SPLScar scratcher (201907, SPL Life Sciences, Pocheon, Republic of Korea), and cells were incubated with trophoblast-derived CM for 24 h. Cell migration was recorded using an Olympus IX71 microscope (Tokyo, Japan) equipped with a digital camera (Canon Inc., Tokyo, Japan). The wound width was calculated as the average distance between the edges of the scratch using ImageJ software. For the Boyden chamber assay to determine trophoblast invasion, CM were placed in the lower chambers of Transwell plates with 6.5 mm diameter polycarbonate filters (8-μm pore size; Corning Inc., Corning, NY, USA), and HTR-8/SVneo cells (5×10^3 cells) were placed into the upper wells, which were pre-coated with 100 μl of growth factor-reduced Matrigel (354230, Corning Inc.). After incubation for 4 h in a humidified CO_2 incubator, cells were carefully fixed with cold methanol (4°C) and stained with crystal violet, and those remaining on the upper surface of each filter were wiped off using a cotton swab. The cells found on the lower side of the filter were observed under an Olympus IX71 microscope, and images were captured using a video graphic system. Cell invasion was quantified by counting the cells in all fields in each assay.

Histology and immunohistochemistry

Paraffin-embedded kidney and placental tissues were sectioned at a thickness of 5 μm using a cryostat microtome (CM1850 UV, Leica Biosystems, Wetzlar, Germany). Sections of renal tissue were stained with H&E, followed by analysis under an Olympus IX71 microscope. The mean glomerular diameter was calculated from five glomeruli that were randomly selected from the renal tissue of each mouse. Bowman's capsule space was calculated by subtracting the glomerular tuft area from the renal corpuscle area using ImageJ. The sections of placental tissues were stained with H&E as well as an anti-

cytokeratin antibody (ab9377, Abcam, Cambridge, MA, USA; 1:100) and IgG-Alexa Fluor 488 (A21206, Invitrogen; 1:400) for trophoblast invasion assessment, an anti-CD31 antibody (MA3105, Invitrogen; 1:100) and IgG-Alexa Fluor 647 (ab173004, Abcam; 1:400) for endothelial cell analysis, or an anti-α-SMA antibody (M0851, Dako, Carpinteria, CA, USA; 1:100) and IgG-Alexa Fluor 555 (A21427, Invitrogen; 1:400) for vascular smooth muscle cell analysis. Thereafter, the sections were stained with 4′,6-diamidino-2-phenylindole dihydrochloride (DAPI, 1 mg/ml) for 30 min in the dark. Images of the slides were captured using a confocal laser scanning microscope (LSM-880, Carl Zeiss, Oberkochen, Germany). The fluorescent area in each vessel was quantified and normalized to the total vessel area using the ZEN software (Carl Zeiss).

References

1. Schulz, L. C. & Widmaier, E. P. The effect of leptin on mouse trophoblast cell invasion. *Biol. Reprod.* **71**, 1963–1967 (2004).

2. Li, L. & Schust, D. J. Isolation, purification and in vitro differentiation of cytotrophoblast cells from human term placenta. *Reprod. Biol. Endocrinol.* **13**, 71 (2015).

3. Kobayashi, M. *et al.* A simple method of isolating mouse aortic endothelial cells. *J. Atheroscler. Thromb.* 12, 138–142 (2005).

4. Kim, S. *et al.* Circulating miRNAs associated with dysregulated vascular and trophoblast function as target-based diagnostic biomarkers for preeclampsia. *Cells* **9**, 2003 (2020).

5. Kim, J. *et al.* Aspirin prevents TNF-α-induced endothelial cell dysfunction by regulating the NF-κB-dependent miR-155/eNOS pathway: Role of a miR-155/eNOS axis in preeclampsia. *Free. Radic. Biol. Med.* **104**, 185–198 (2017).

Supplementary Table 1. List of qRT−PCR primers

Genes	Forward primer sequence	Reverse primer sequence
hsa-pre-*miR-214*	CTGGACAGAGTTGTCATGTGT	GTCATTCAGGCTGGGTTGTC
h*eNOS*	GTGGCTGTCTGCATGGACCT	CCACGATGGTGACTTTGGCT
h*PlGF*	GTTCAGCCCATCCTGTGTCT	TTAGGAGCTGCATGGTGACA
h*GAPDH*	CCACCCATGGCCAAATTCCATGGCA	TCTAGACGGCAGGTCAGGTCCACC
mmu-pre-*miR-214*	GTCTGCCTGTCTACACTTGC	CTTTCAATGGCTGGTGGTCA
m*eNOS*	AGAGATTGGCATGAGGGACC	ATTAATTTCCACCGCTGCCT
m*PlGF*	TGAAGGCATGTAGAGGGGAC	CACTCTGCCTGTGTTCCAGA
m*Twist1*	GGACAAGCTGAGCAAGATTCA	GAGTCGATGCGGAAGAGGC
sgRNA	ATAGGCCTGTACAGCAGGCACAGACG	AAAACGTCTGTGCCTGCTGTACAGGC
m*GAPDH*	AGCGACCCCTTCATTGAC	TCCACGACATACTCAGCAC

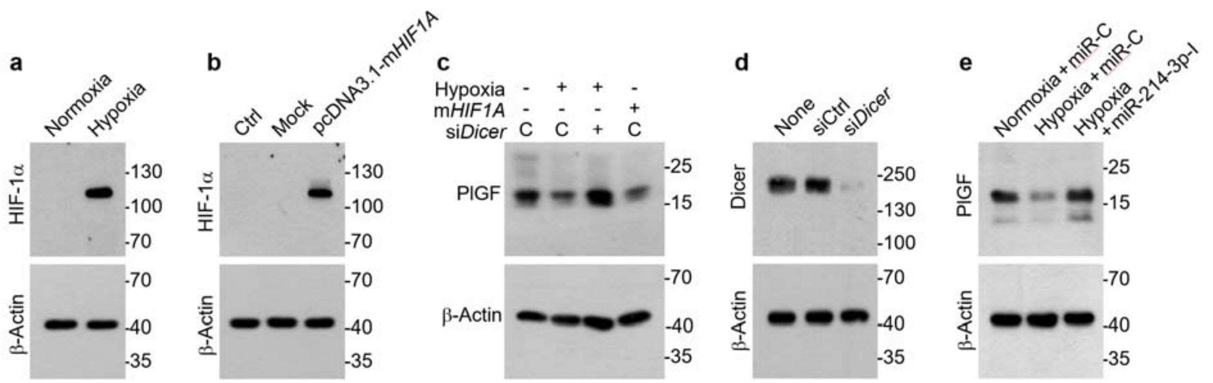

Supplementary Fig. 1. Levels of PlGF, HIF-1α, and Dicer in trophoblasts under various experimental conditions. **a**, **b** HIF-1α levels were determined in HTR-8/SVneo cells exposed to normoxia or hypoxia for 24 h or transfected with mock or pcDNA3.1-mutant *HIF1A* (m*HIF1A*) for 24 h using western blotting. **c** PlGF levels were determined in HTR-8/SVneo cells exposed to normoxia or hypoxia after transfection with mock, pcDNA3.1-mutant *HIF1A* (m*HIF1A*), siControl (C), or si*Dicer* (si*Dicer*) using western blotting. **d** Dicer levels were determined in HTR-8/SVneo cells transfected with siControl or si*Dicer* for 24 h using western blotting. **e** PlGF levels were determined using western blotting in HTR-8/SVneo cells exposed to normoxia or hypoxia after transfection with control miRNA (miR-C) or an miR-214-3p inhibitor (miR-214-3p-I).

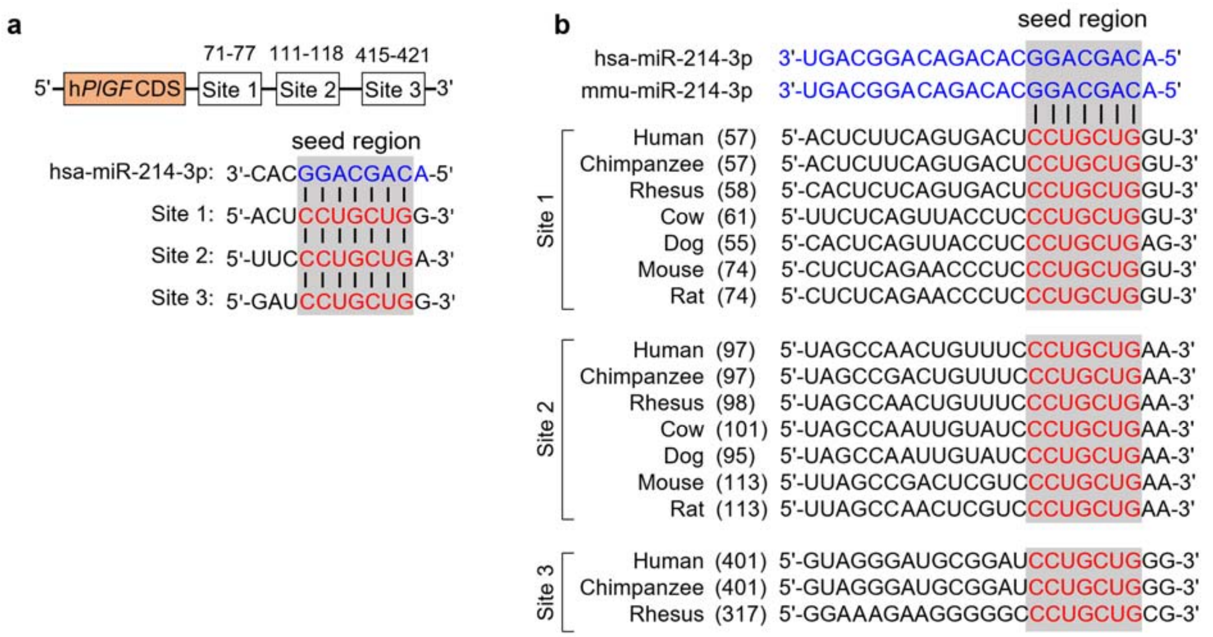

Supplementary Fig. 2. Computational prediction of miR-214-3p targets in *PlGF* 3′-UTRs of various species. a The computational analysis using the TargetScan algorithm shows three putative complementary binding regions (71–77, 111–118, and 415–421 nt) between human miR-214-3p and human *PlGF* 3′-UTR. h*PlGF* CDS, coding DNA sequence of human *PlGF* gene. **b** Complementary binding nucleotide sequence alignment between human or mouse miR-214-3p (hsa- and mmu-miR-214-3p) and *PlGF* 3′-UTRs of human, nonhuman primates, mouse, rat, and other species.

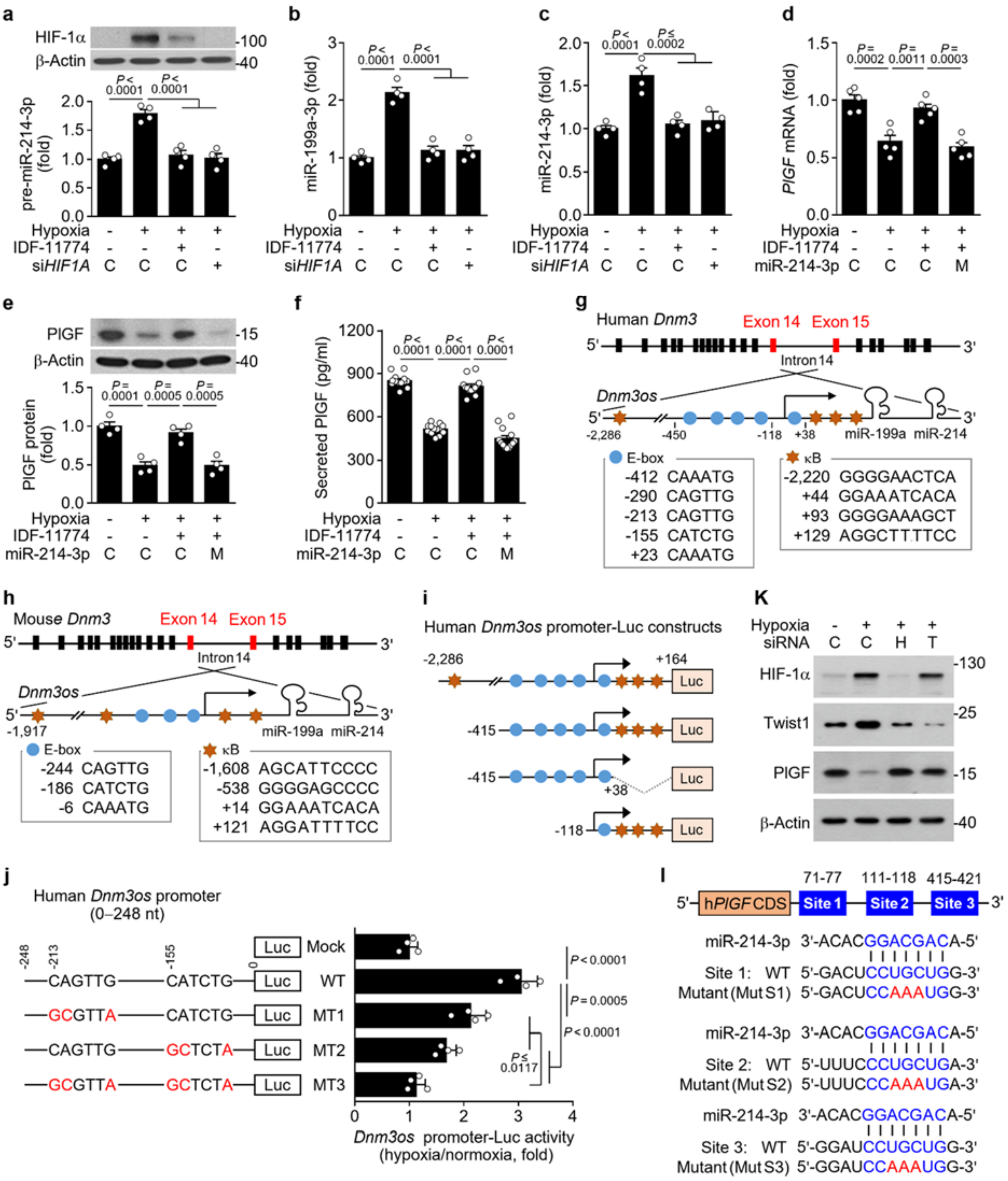

Supplementary Fig. 3. Hypoxia decreases PlGF expression through HIF-1/Twist1-dependent miR-214-3p biogenesis. **a**–**f** HTR-8/SVneo cells (**a**, **b**) or BeWo cells (**c**–**f**) were transfected with siRNA for control (C) or *HIF1A* (si*HIF1A*), control miRNA (C), or miR-214-3p mimics (M), followed by exposure to normoxia or hypoxia in the presence or absence of IDF-11774 for 24 h. **a**, **b** The levels of pre-miR-214-3p (**a**) and miR-199a-3p (**b**) were determined in HTR-8/SVneo cells using qRT–PCR (n = 4). **c**–**f** The levels of miR-214-3p (n = 4, **c**), *PlGF* mRNA (n = 5, **d**), PlGF protein (n = 4, **e**), and secreted PlGF (n = 13, **f**) were determined in BeWo cells using qRT–PCR, western blotting, or ELISA.

g, **h** Computational analysis of the putative transcription factor-binding sites, E-box and κB, in the promoter regions of human (**g**) and mouse miR-199a/214 (*Dnm3os*) (**h**). **i** Schematic plasmid (pGL3) constructs containing WT, truncated, or deleted promoter of human miR-199a/214 (*Dnm3os*). **j** HTR-8/SVneo cells were transfected with WT or mutant pGL3-*Dnm3*os promoter and exposed to normoxia or hypoxia for 24 h. Luciferase reporter activities were determined in cell lysates using an enzyme assay kit ($n = 4$). **k** HTR-8/SVneo cells were transfected with siRNA for control (C), *HIF1A* (H), or *Twist1* (T), followed by exposure to normoxia or hypoxia for 24 h. The levels of target proteins were determined using western blotting. **l** Schematic illustration of psiCHECK-2 luciferase constructs containing WT or mutants at site 1 (71–77 nt, Mut S1), site 2 (111–118 nt, Mut S1), and site 3 (415–421 nt, Mut S3) of human *PlGF* 3′-UTR (876 bp). Data are presented as the mean ± SEM. Statistical significance was determined using one-way ANOVA (**a**–**f**, **j**), followed by the Holm–Sidak′s multiple comparisons test.

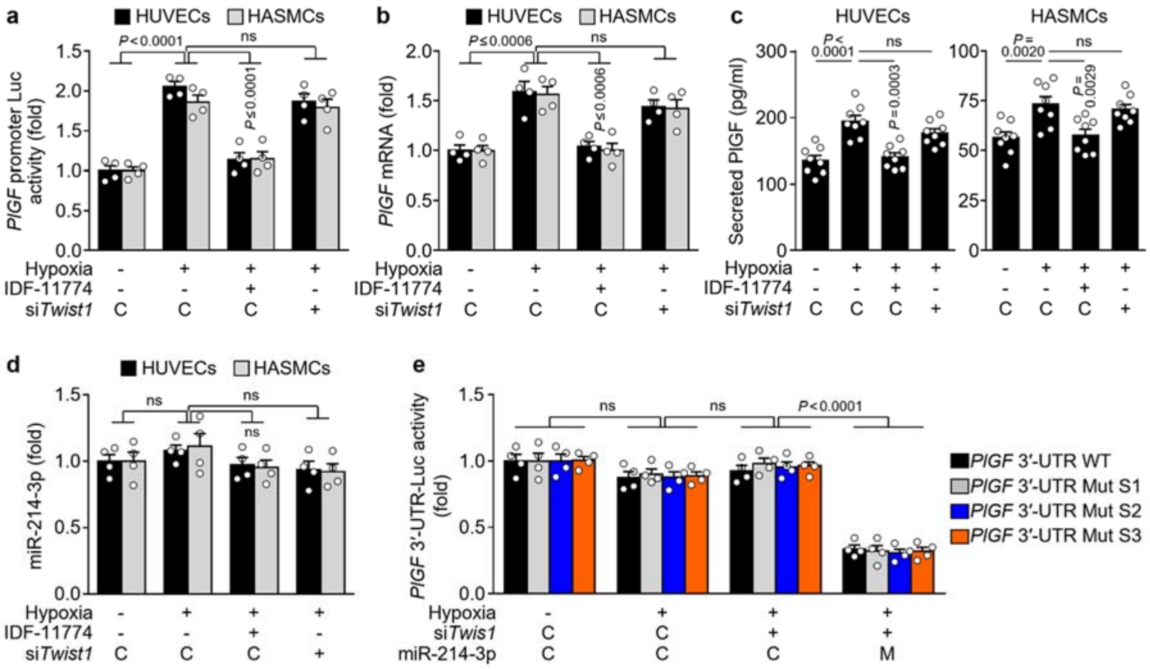

Supplementary Fig. 4. Hypoxia increases PlGF expression but does not promote miR-214-3p biogenesis in HUVECs and HASMCs. a HUVECs and HASMCs were transfected with the pGL3-*PlGF* promoter in combination with siRNA for control (C) or *Twist1*, followed by exposure to normoxia or hypoxia in the presence or absence of IDF-11774 for 24 h. Luciferase reporter activity was determined using an enzyme assay kit (n = 4). **b–d** Both types of vascular cells were treated as in (**a**) without transfection with the pGL3-*PlGF* promoter. The levels of *PlGF* mRNA (n = 4, **b**), secreted PlGF (n = 8, **c**), and miR-214-3p (n = 4, **d**) were determined using qRT–PCR or ELISA. **e** HUVECs were transfected with psiCHECK-2-WT or mutant *PlGF* 3′-UTRs, control siRNA (C), *Twist* siRNA, control miRNA (C), or miR-214-3p mimics (M), followed by exposure to normoxia or hypoxia for 24 h. Luciferase activity was measured using an enzyme assay kit (n = 4). Data are presented as the mean ± SEM. Statistical significance was determined using one-way (**a–d**) or two-way ANOVA (**e**), followed by the Holm–Sidak′s multiple comparisons test. ns, not significant.

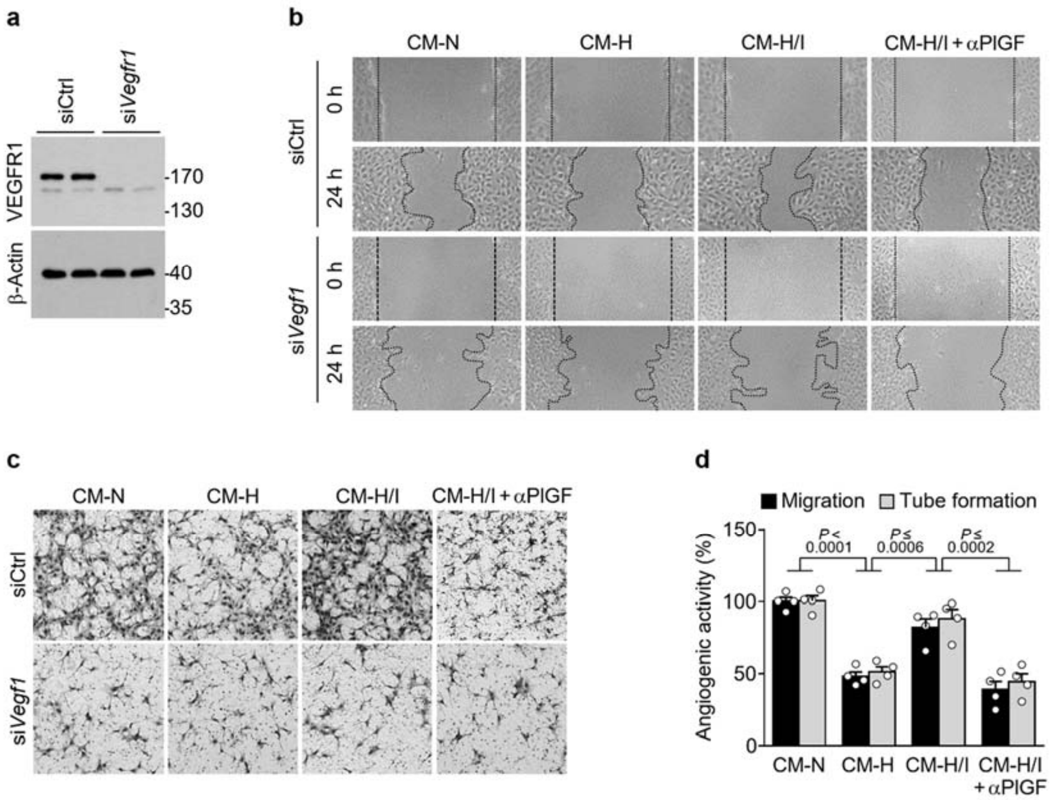

Supplementary Fig. 5. Hypoxia suppresses trophoblast function through miR-214-3p-dependent PlGF downregulation. a VEGFR1 levels were assessed in HTR-8/SVneo cells transfected with *Vegfr1* siRNA using western blotting. **b**, **c** Representative images of the scratch-wound assay (**b**) and Boyden chamber assay (**c**) on HTR-8/SVneo cells incubated in CM-N, CM-H, CM-H/I, or CM-H/I + anti-PlGF antibody (αPlGF) following transfection with control or *Vegfr1* siRNA. **d** Angiogenic activities of CM-N, CM-H, CM-H/I, or or CM-H/I + αPlGF were quantitated in cultured HUVECs using ImageJ software (n = 4). CM-N, CM-H, and CM-H/I were prepared using HTR-8/SVneo cells exposed to normoxia, hypoxia, and hypoxia with an miR-214-3p inhibitor treatment, respectively. Data are presented as the mean ± SEM. Statistical significance was determined using one-way ANOVA (**d**), followed by the Holm–Sidak′s multiple comparisons test.

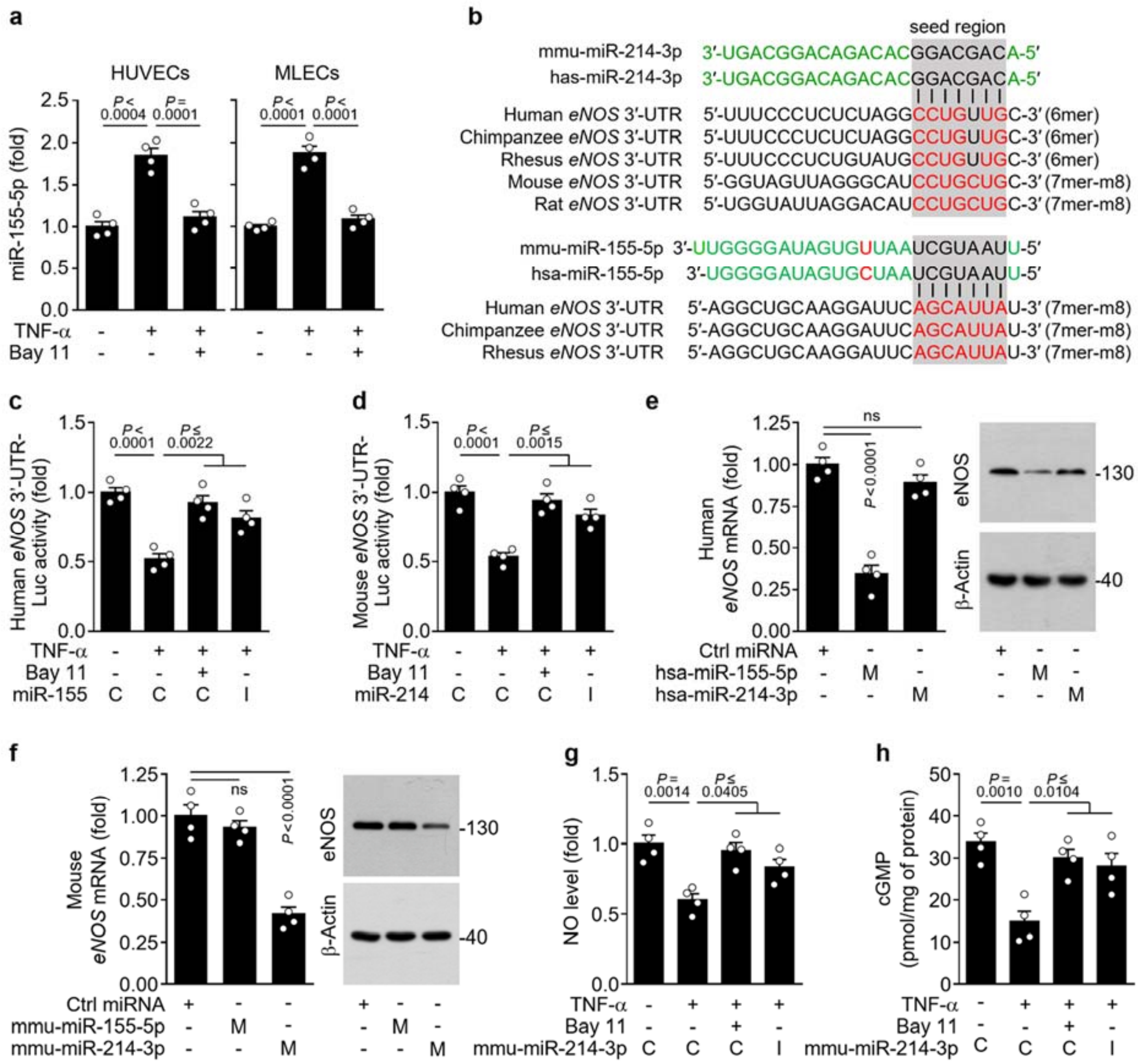

Supplementary Fig. 6. TNF-α suppresses eNOS expression in endothelial cells by elevating NF-κB-responsive miR-155-5p or miR-214-3p biogenesis in a species-specific manner. a HUVECs and MLECs were treated with TNF-α or/and Bay 11-7082 (Bay 11), followed by measurement of miR-155-5p levels using qRT–PCR (n = 4). **b** Computational analysis of putative complementary binding sequences for miR-214-3p and miR-155-5p in *eNOS* 3′-UTRs of various species using the TargetScan algorithm. **c**, **d** HUVECs (**c**) and MLECs (**d**) were transfected with psiCHECK-2-human or mouse *eNOS* 3′-UTRs as well as control miRNA or an miR-214 or miR-155-5p inhibitor (I), followed by treatment with TNF-α or/and Bay 11-7082. Luciferase activity was determined using an enzyme assay kit (n = 4). **e–h** HUVECs (**e**) and MLECs (**f–h**) were transfected with control miRNA (C), or human or mouse synthetic miR-155-5p or miR-214 inhibitor (I), followed by determination of eNOS mRNA and protein levels (**e**, **f**), NO production (**g**), and cGMP synthesis (**h**) using qRT–PCR, western blotting, DAF-FM-based fluorescence microscopy, or ELISA (n = 4). Data are presented as the mean ± SEM. Statistical significance was determined using one-way ANOVA (**a**, **c–h**), followed by the Holm–Sidak′s multiple comparisons test. ns, not significant.

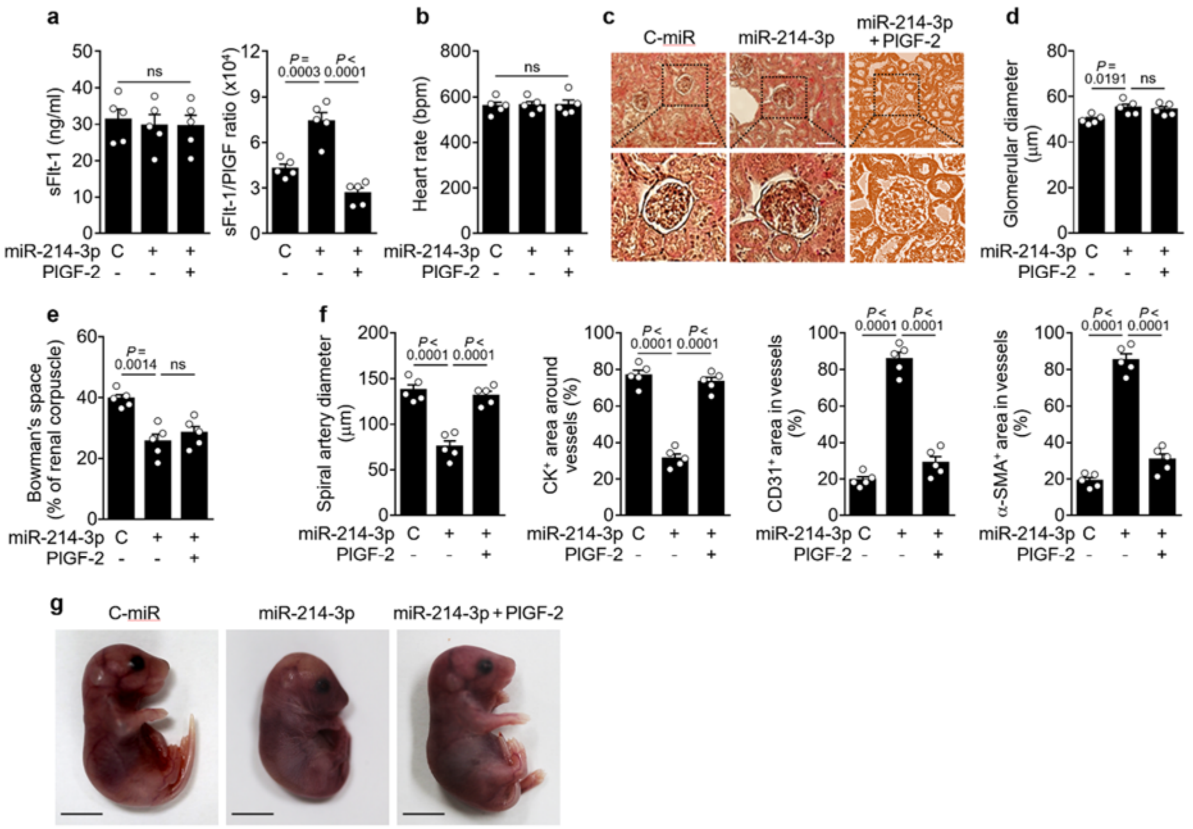

Supplementary Fig. 7. Exogenous miR-214-3p induces PE-like phenotypes in pregnant mice. Pregnant mice were infused with locked nucleic acid-based control miRNA (C), synthetic miR-214-3p mimics (miR-214-3p), or miR-214-3p plus rmPlGF-2 (PlGF-2) from GD 9.5 to GD 17 using an osmotic pump. **a** Circulating sFlt-1 levels were determined using an ELISA kit (n = 5), and the sFlt-1/PlGF ratio was calculated from the plasma concentrations of each protein (n = 5). **b** Heart rate was determined at GD 17 using a noninvasive tail-cuff method (n = 5). **c** Representative images of H&E-stained maternal renal tissues. Scale bar = 50 μm. **d** Glomerular diameter was calculated using ImageJ and expressed as the average value of five random glomeruli in each renal tissue section (n = 5). **e** Bowman's capsule space was calculated by subtracting the glomerular tuft area from the renal corpuscle area (n = 5). **f** Diameter of spiral arteries was measured using ImageJ and expressed as the average value of five random vessels in each placental tissue section. Areas of CK^+ trophoblasts, $CD31^+$ endothelial cells, and α-SMA^+ smooth muscle cells around or in vessels were determined using the ZEN software and expressed as the mean value of five random vessels in each placental tissue section (n = 5). **g** Representative photographs of GD 17.5 fetuses. Scale bar = 0.5 cm. Data are presented as the mean ± SEM. Statistical significance was determined using one-way ANOVA (**a**, **b**, **d**–**f**), followed by the Holm–Sidak's multiple comparisons test. ns, not significant.

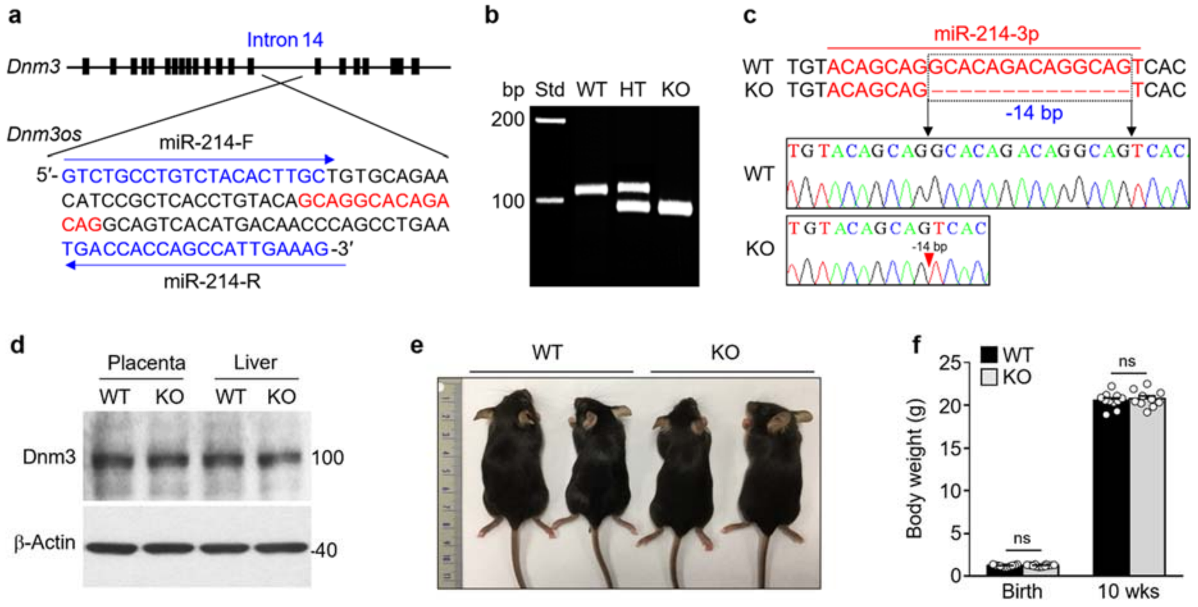

Supplementary Fig. 8. Generation of miR-214-3p KO mice. a Gene structure of mouse *Dnm3* and nucleotide sequence of the PCR-amplified region encompassing the CRISPR target in mouse *Dnm3os*. Fourteen nucleotides marked in red were deleted using CRISPR-Cas9. MiR-214-F and miR-214-R in blue color indicate the primers used for PCR amplification. **b** Detection of PCR products (107 bp for WT mice and 93 bp for miR-214-3p KO mice) amplified using genomic DNA from WT and miR-214-3p KO mice. HT, heterozygous. **c** Sequencing chromatograms of PCR amplicons from WT and miR-214-3p KO mice. The red arrow above the chromatograms indicates the deletion of 14 nucleotides. **d** Dnm3 levels determined in placental and liver tissues using western blotting. **e** Representative photographs of WT and miR-214-3p KO mice at 8 weeks of age. **f** Body weight of newborn and 10-week-old mice (n = 10–12). Data are presented as the mean ± SEM. Statistical significance was determined using the unpaired two-tailed t test. ns, not significant.

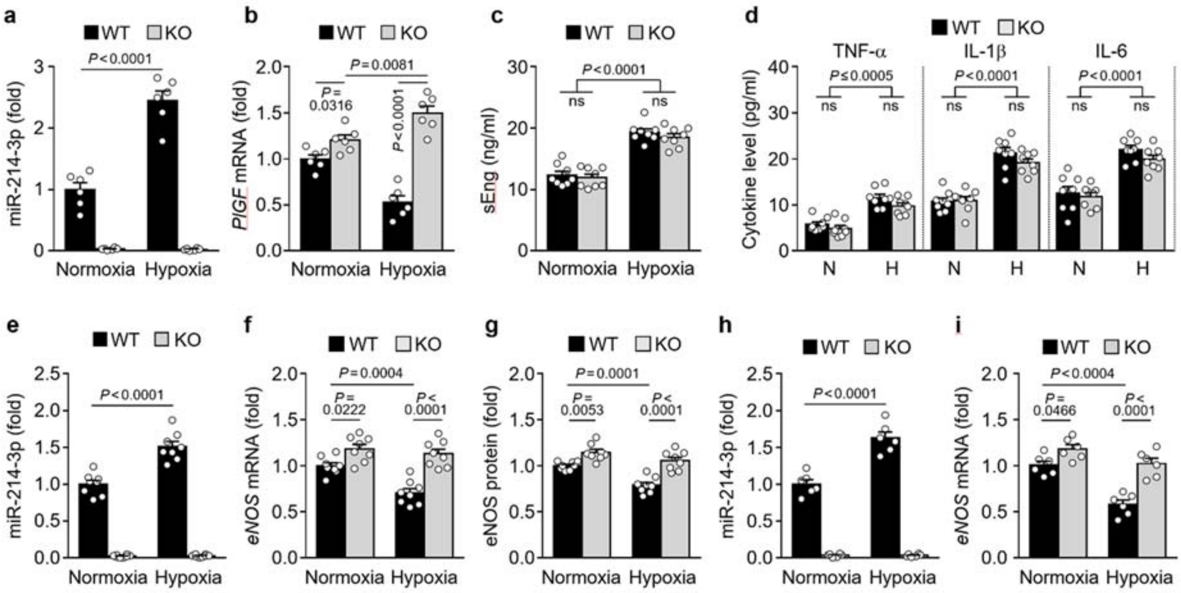

Supplementary Fig. 9. MiR-214-3p deficiency does not alter sEng and inflammatory cytokine levels and rescues eNOS expression in hypoxic pregnant mice. Pregnant WT and miR-214-3p KO mice were exposed to normoxia (N) or hypoxia (H) from GD 9.5 to GD 17.5. **a**, **b** MiR-214-3p (**a**) and *PlGF* mRNA levels (**b**) were determined in mouse primary trophoblasts using qRT–PCR (n = 6). **c** Serum levels of sEng were quantified using ELISA kits (n = 8). **d** The serum levels of TNF-α, IL-1β, and IL-6 were determined using ELISA kits (n = 8). **e–g** The levels of miR-214-3p (e), eNOS mRNA (**f**) and protein (**g**) in aortic vessels were determined using qRT–PCR and western blotting (n = 8). **h**, **i** Level of miR-214-3p and eNOS mRNA were determined in mouse aortic endothelial cells using qRT–PCR (n = 6). Data are presented as the mean ± SEM. Statistical significance was determined using one-way ANOVA, followed by the Holm–Sidak's multiple comparisons test. ns, not significant.

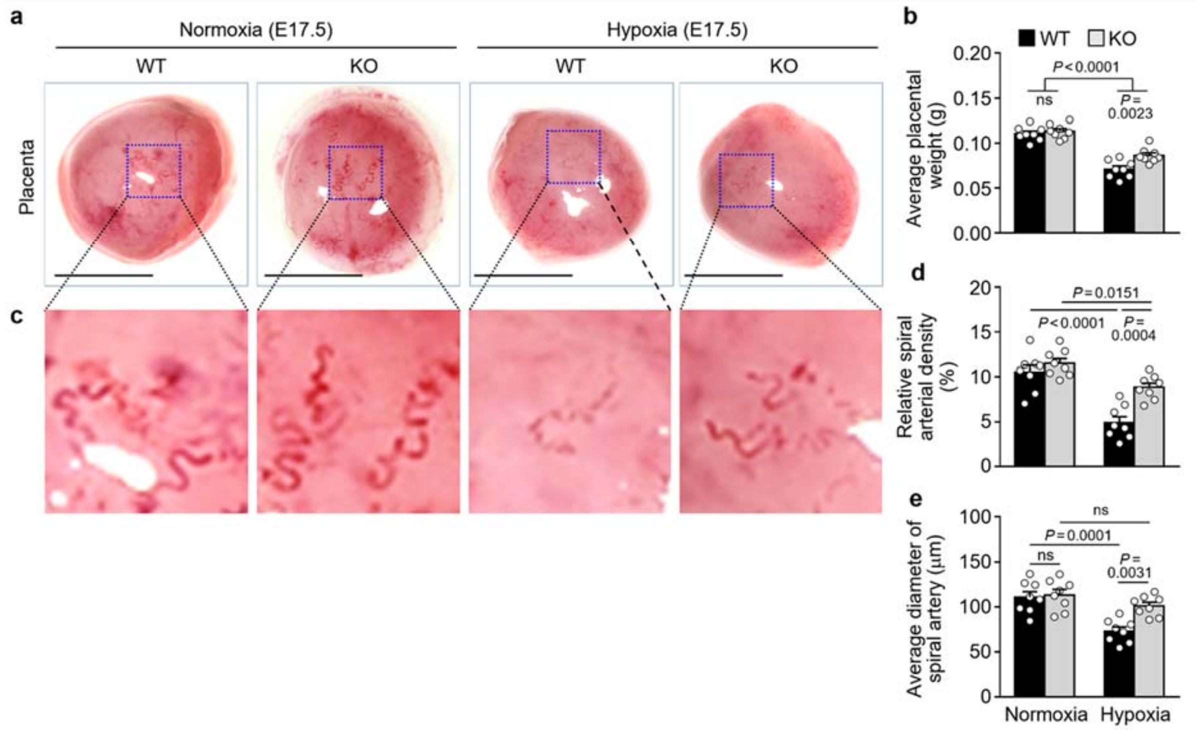

Supplementary Fig. 10. MiR-214-3p deficiency improves placental growth and arterial abnormality in hypoxic pregnant mice. a Representative images of placentas from pregnant WT and miR-214-3p KO mice exposed to normoxia or hypoxia from GD 9.5 to GD 17.5 (6–11 fetuses/litter, *n* = 8 litters). Scale bar = 5 mm. **b** Quantification of the average placental weight/litter (*n* = 8 litters). **c** Magnified images of maternal spiral arteries of the placental bed (*n* = 8 litters). **d** The relative density of the decidual spiral arteries in the placental bed was calculated using ImageJ (*n* = 8 litters). **e** The average diameter of the spiral arteries was calculated using ImageJ (*n* = 8 litters). Data are presented as the mean ± SEM. Statistical significance was determined using one-way ANOVA (**b**, **d**, **e**), followed by the Holm–Sidak′s multiple comparisons test. ns, not significant.

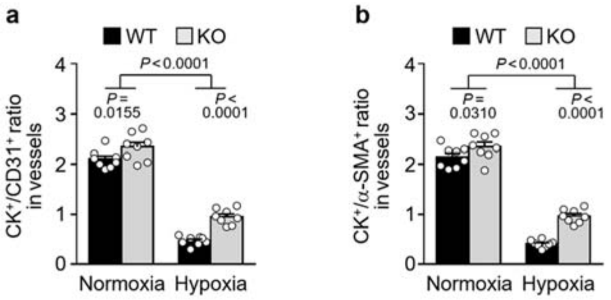

Supplementary Fig. 11. MiR-214-3p deficiency improves spiral artery remodeling by trophoblast invasion in hypoxic pregnant mice. Pregnant WT and miR-214-3p KO mice were exposed to normoxia or hypoxia from GD 9.5 to GD 17.5. **a** Quantification of the cytokeratin $(CK)^+$ trophoblast area to the $CD31^+$ endothelial cell area ratio in spiral arteries from immunohistochemical data shown in Fig. 8 (6–11 fetuses/litter, n = 8 litters). **b** Quantification of the CK^+ trophoblast area to the $\alpha\text{-}SMA^+$ smooth muscle cell area ratio in spiral arteries from immunohistochemical data shown in Fig. 8 (n = 8 litters). Data are presented as the mean ± SEM. Statistical significance was determined using one-way ANOVA, followed by the Holm–Sidak′s multiple comparisons test.

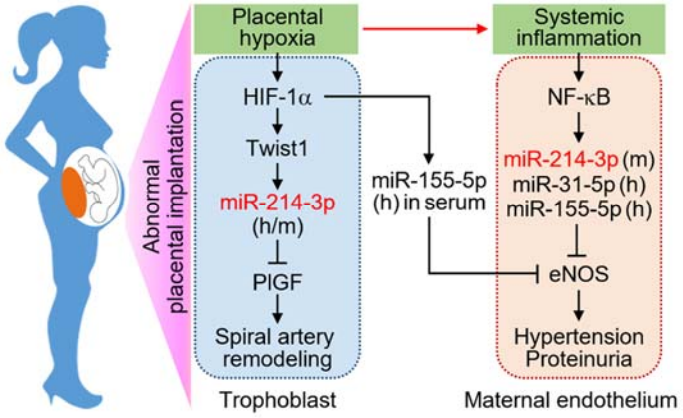

Fig. 12. Schematic diagram of the putative role of hypoxia- and inflammation-responsive miR-214-3p, miR-31-5p, and miR-155-5p in PE pathogenesis. Abnormal or inappropriate placental implantation causes placental hypoxia, which stimulates immune cell activation and proinflammatory cytokine expression and secretion into maternal circulation, causing systemic inflammation. Placental hypoxia increases miR-214-3p biogenesis in trophoblasts in an HIF-1/Twist1-dependent manner and reduces PlGF expression, impairing spiral artery remodeling. Meanwhile, systemic inflammation upregulates NF-κB-responsive miR-214-3p (h, human; m, mouse), miR-31-3p (h, human), and miR-155-5p (h, human) expression and inhibits eNOS expression and NO/cGMP synthesis, resulting in maternal hypotension and proteinuria. Placental hypoxia can also induce miR-155-5p (h, human) biogenesis and secretion into maternal circulation in an exosome-associated form, leading to maternal endothelial dysfunction through downregulation of eNOS expression.
